# Increase of Radiative Forcing through Midinfrared Absorption by Stable CO_2_ Dimers?

**DOI:** 10.1021/acs.jpca.2c00857

**Published:** 2022-05-09

**Authors:** Dennis F. Dinu, Pit Bartl, Patrick K. Quoika, Maren Podewitz, Klaus R. Liedl, Hinrich Grothe, Thomas Loerting

**Affiliations:** † Institute of General, Inorganic and Theoretical Chemistry, University of Innsbruck, A-6020 Innsbruck, Austria; ‡ Institute of Physical Chemistry, University of Innsbruck, A-6020 Innsbruck, Austria; § Institute of Materials Chemistry, Technische Universität Wien, A-1060 Vienna, Austria

## Abstract

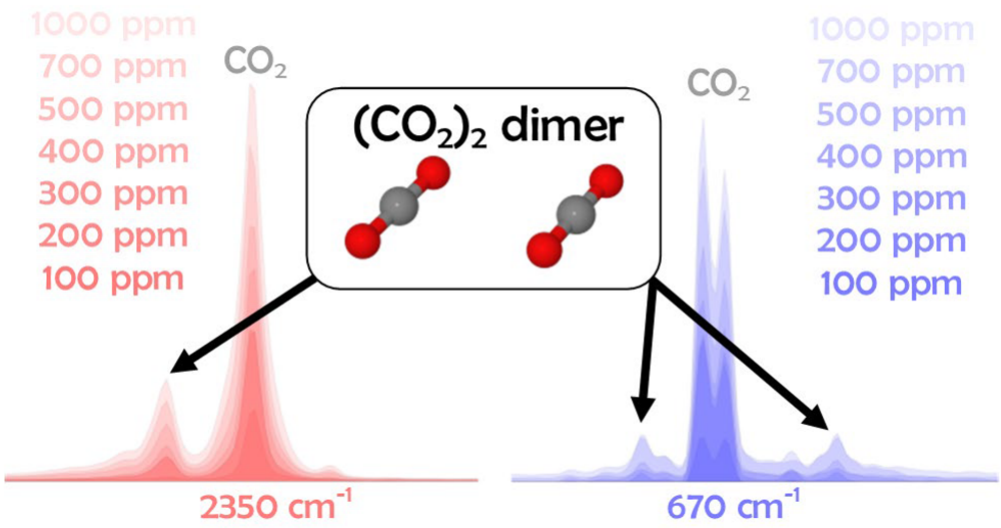

We performed matrix-isolation infrared (MI-IR) spectroscopy of carbon dioxide monomers, CO_2_, and dimers, (CO_2_)_2_, trapped in neon and in air. On the basis of vibration configuration interaction (VCI) calculations accounting for mode coupling and anharmonicity, we identify additional infrared-active bands in the MI-IR spectra due to the (CO_2_)_2_ dimer. These bands are satellite bands next to the established CO_2_ monomer bands, which appear in the infrared window of Earth’s atmosphere at around 4 and 15 μm. In a systematic carbon dioxide mixing ratio study using neon matrixes, we observe a significant fraction of the dimer at mixing ratios above 300 ppm, with a steep increase up to 1000 ppm. In neon matrix, the dimer increases the IR absorbance by about 15% at 400 ppm compared to the monomer absorbance alone. This suggests a high fraction of the (CO_2_)_2_ dimer in our matrix experiments. In atmospheric conditions, such increased absorbance would significantly amplify radiative forcings and, thus, the greenhouse warming. To enable a comparison of our laboratory experiment with various atmospheric conditions (Earth, Mars, Venus), we compute the thermodynamics of the dimerization accordingly. The dimerization is favored at low temperatures and/or high carbon dioxide partial pressures. Thus, we argue that matrix isolation does not trap the gas composition “as is”. Instead, the gas is precooled to 40 K, where CO_2_ dimerizes before being trapped in the matrix, already at very low carbon dioxide partial pressures. In the context of planetary atmospheres, our results improve understanding of the greenhouse effect for planets of rather thick CO_2_ atmospheres such as Venus, where a significant fraction of the (CO_2_)_2_ dimer can be expected. There, the necessity of including the mid-IR absorption by stable (CO_2_)_2_ dimers in databases used for modeling radiative forcing, such as HITRAN, arises.

## Introduction

Carbon dioxide is the preeminent anthropogenic greenhouse gas on Earth.^1^ In thermal equilibrium with the sun, the Earth’s surface temperature should be 255 K according to Stefan–Boltzmann’s law. The actual surface temperature, however, is 288 K, implying a warming of 33 K caused by its atmospheric greenhouse gases. While CO_2_ is a trace gas on Earth, Venus has a much denser atmosphere composed of mainly 96.5% CO_2_ and 92 bar of surface pressure.^2^ Hence, the greenhouse effect on Venus is about 15 times stronger than that on Earth. The Earth’s atmospheric CO_2_ mixing ratio increased from 270 ± 10 ppm in 1750 to about 417 ppm in May 2020, where annual increases have reached 3 ppm in the past decade.^3^ Consequently, the temperature has risen by +2.50 ± 0.14 °C for Northern hemisphere landmasses comparing 2020 with 1884.^4^ By year 2100, CO_2_ mixing ratios might climb to 830 ppm according to the SSP3 scenario that predicts an increase of global mean surface temperature by 4.1 °C,^5^ far above the Paris Agreement aims of 1.5 °C.^6^ This scenario seems to be a path closely resembling that of the global development in the past few years.^7^

A skyrocketing atmospheric CO_2_ mixing ratio favors CO_2_ dimerization and raises the question of what the impact of (CO_2_)_2_ on greenhouse warming might be. Up to now, no carbon dioxide dimers have been detected in Earth’s atmosphere. However, once dimers appear in significant amounts in planetary atmospheres, we expect increasing radiative forcing due to additional infrared (IR) absorption and, thus, global warming beyond the greenhouse effect of the monomers. If there were carbon dioxide dimers in the troposphere, revision of contemporary climate models could be necessary because of potentially unaccounted (CO_2_)_2_ infrared absorptions. A step in this direction has been done by considering collision-induced absorption (CIA) due to unstable (CO_2_)_2_ dimers for dense CO_2_ atmospheres.^8^ CIA has been included in the HITRAN database for the (CO_2_)_2_ dimers, together with a variety of other dimers and other collision complexes.^9^ However, dimerization may also result in stable dimers (or tightly bound dimers), which have been recently considered in broad-band spectra.^10^ In the mid-IR, such stable dimers would lead to permanent additional absorptions in the vicinity of the monomer’s IR absorption. Those additional IR bands are usually hard to distinguish from the rotational–vibrational spectrum of the pure monomer.

Thus, we investigate the IR spectrum of carbon dioxide using matrix isolation to quench rotational transitions and use anharmonic calculations to assign all observed vibrational transitions. Previously, we successfully assigned all CO_2_ monomer bands,^11^ where we also provided a short review of the theoretical and experimental spectroscopy of carbon dioxide and its dimer. For a detailed review on computational studies on the (CO_2_)_2_ dimer and also recent calculations we refer to Maystrovsky et al.,^12^ who demonstrated that variational calculations using a tailor-made potential energy surface for the dimer on explicitly correlated coupled cluster theory yield excellent results. In the present work, we investigate the infrared absorption of the (CO_2_)_2_ dimer in the mid-IR and discuss its importance to different planetary atmospheres, specifically Mars, Venus, and Earth in comparison. On the basis of two matrix-isolation experiments, we demonstrate the characteristics of the mid-IR spectrum of the (CO_2_)_2_ dimer: (a) a mixing ratio series study of carbon dioxide isolated in neon matrix and (b) direct matrix isolation of laboratory air (containing about 417 ppm carbon dioxide). In addition to the experimental data, we provide *ab initio* calculations of the vibrational spectrum of the monomer and dimer, relying on multimode potential energy surfaces^13^ and vibrational self-consistent field (VSCF) and configuration interaction (VCI) computations.^14,15^ This approach allows us to unequivocally identify dimer absorptions by minimizing the discrepancy between experiment and calculation based on the inclusion of mode coupling and anharmonicity.^16^ Finally, we calculate the equilibrium constant for the monomer–dimer equilibrium to estimate the fraction of dimers for different CO_2_ partial pressures and temperatures.

## Experimental Methodology and Computational Details

### Matrix-Isolation Infrared Spectroscopy of Carbon Dioxide in Neon

We performed infrared spectroscopy measurements of mixtures of gases in frozen neon matrixes, i.e., matrix-isolation infrared (MI-IR) spectroscopy. Former studies^17,18^ have shown that the composition of an atmosphere trapped in a frozen matrix resembles the gas phase composition. Thus, MI-IR spectroscopy is a suitable analytical technique for investigating short-lived and unstable species from the gas phase. As matrix isolation inhibits molecular rotation, i.e., quenches the rotational–vibrational transitions, the complexity of the MI-IR spectra is reduced compared to a gas-phase IR spectrum. Consequently, observation of pure vibrational features in MI-IR spectra facilitates the distinction of different molecules, conformers, and clusters as well as dimers and oligomers. For studying the carbon dioxide dimerization with increasing carbon dioxide mixing ratio, we employed MI-IR experiments using neon as a host. Compared to other rare gases as a matrix material, neon minimizes matrix effects such as matrix shifts or multiple trapping sites.^11^ Hence, MI-IR spectroscopy using neon as a host provides particularly clean spectra and facilitates studying carbon dioxide dimerization.

We used a ^12^C^16^O_2_ sample of high purity (99.9995%, Messer Austria, order no. 1290102114, lot 27531923) to deposit CO_2_/Ne mixtures as a frozen, immobile solid on a cold mirror. The neon matrix containing carbon dioxide is then probed in the mid-infrared using a Vertex 80v Bruker spectrometer. The mixing ratio of carbon dioxide in neon, from now on denoted as ρ, was adjusted and quantified by barometric monitoring. This setup is detailed elsewhere.^11,19^ In our main experiments, matrix isolation and IR spectroscopy were performed at 6 K. We accumulated the IR spectra (resolution, 0.3 cm^–1^; scans, 512) within roughly 30 min. To study the influence of initial gas temperature during mixing on the dimerization, the CO_2_/Ne mixtures were equilibrated at either 25 °C (cf. the spectra in Figure S4) or 65 °C (cf. spectra in Figure S5). The relative band areas for the monomer and dimer at these different mixing temperatures (cf. Figure S6, parts b and c) show no significant difference.

As neon has a comparably high vapor pressure in comparison to, e.g., argon, we probed the thermal stability of our neon matrixes. All gas mixtures were deposited at 6 K, and then slowly heated in steps of 0.5 K up to 12 K. In each step, we accumulated “quick” spectra (resolution, 0.3 cm^–1^; scans, 32) within about 1 min. Neon evaporation from the matrix commences at roughly 9 K. Afterward we further increased the temperature beyond 12 K by turning off the helium cryostat and keeping the heater on. In roughly 2 min steps, we accumulated spectra up to a temperature of about 100 K. Figure S7 shows for ρ = 500 ppm that solid carbon dioxide residues remain after neon evaporation. We observed very similar behavior for the other carbon dioxide mixing ratios in our study.

### MI-IR Spectroscopy of Isolated Air (Carbon Dioxide in Nitrogen–Oxygen–Argon)

When isolating air at 12 K, all trace gases in the air, including carbon dioxide, are trapped in a solid matrix of N_2_/O_2_/Ar, with their volume mixing ratio of 78:21:1 in the atmosphere. For this experiment, we used a different high-vacuum setup, also employing a helium cryostat. The cryostat was used to cool a cesium iodide window to 12 K. By introducing air into the chamber through a needle valve, we could deposit air onto this window slowly. During the deposition, the window was monitored *in situ*, where the entire vacuum chamber is placed inside the sample chamber of an IR spectrometer (Varian Excalibur 4500). The sample chamber of the spectrometer was constantly flooded with nitrogen to remove IR-active trace gases from the air, through which the beam passes. Over 800 spectra, with a resolution of 0.25 cm^–1^, were collected and combined to a single measurement. We isolated air on November 15, 2020 at Innrain 52c, Innsbruck, Austria. The deposition times were long enough to avoid significant heating of the air matrix.

### Monomer–Dimer Thermodynamic Equilibrium

The free energy of the dimer dissociation (CO_2_)_2_ ↔ 2CO_2_ is computed as Δ*G* (kJ mol^–1^) = 2*G*_CO_2__^f^ – *G*_(CO_2_)_2__^f^, where *G*_(CO_2_)_2__^f^ and *G*_CO_2__^f^ are the free energy of formation of the dimer and the monomer. First, we calculated the electronic energies and the harmonic frequencies of the monomer and dimer at the CCSD(T)-F12 level of theory with the VTZ-F12 basis set using the MOLPRO software package.^20^ Second, we estimated the free energies of formation within the rigid rotor harmonic oscillator (RRHO) approach using the KiSThelP program.^21^ This approach computes thermodynamic properties from partition functions based on previously calculated electronic energies, harmonic frequencies, rotational constants, as well as the molecular symmetry and mass. Note that calculated thermodynamics and the equilibrium constants for temperatures close to 0 K may potentially be inaccurate as the approximations break down. However, for the temperature range we are interested in (above 30 K) the RRHO approximations can be expected to be valid.

### *In Vacuo* VSCF/VCI Calculations of Carbon Dioxide and Its Dimer

Our present band assignment to monomer or dimer vibrational modes relies on VSCF and VCI computations using the MOLPRO software package.^20^ Starting from the global equilibrium structure of the carbon dioxide dimer, which has *C*_2*h*_ symmetry,^22^ we performed a harmonic frequency calculation using the distinguishable cluster approach with a double-ζ basis (DCSD-F12a/VDZ-F12)^23,24^ to obtain the normal modes of the dimer. The multimode potential energy surface (PES) is expanded up to 3D terms, i.e., three mode couplings, using the XSURF algorithm,^25^ as implemented in MOLPRO 2020. Hereby, intermolecular as well as intramolecular modes are coupled. Within this approach we used DCSD-F12a/cc-pVDZ-F12 single points to construct the grid representation of the PES. To estimate whether addition of diffuse functions improves the description of the CO_2_ dimer, we also computed a PES relying on DCSD-F12a/aug-cc-pVDZ single points (cf. Table S2). However, the results were very similar and will not be considered in detail here. The grid representation of the PES is transformed into an analytic representation using polynomials.^26^ On the basis of this polynomial representation, anharmonic vibrational frequencies are computed using the vibrational self-consistent field and configuration interaction (VSCF/VCI) approach^27^ as implemented in MOLPRO. In the VCI approach, single to quadruple (SDTQ) excitations are considered. Vibrational intensities are computed within the same approach using a multimode dipole moment surface (DMS) at the HF/VTZ-F12 level of theory. For the carbon dioxide monomer in *D*_∞*h*_ symmetry, we followed the same procedure using up to three mode couplings. Our previous studies on the CO_2_ monomer using the same computational approach show excellent agreement between VCI computations and MI-IR spectra.^11^

**Figure 1:**
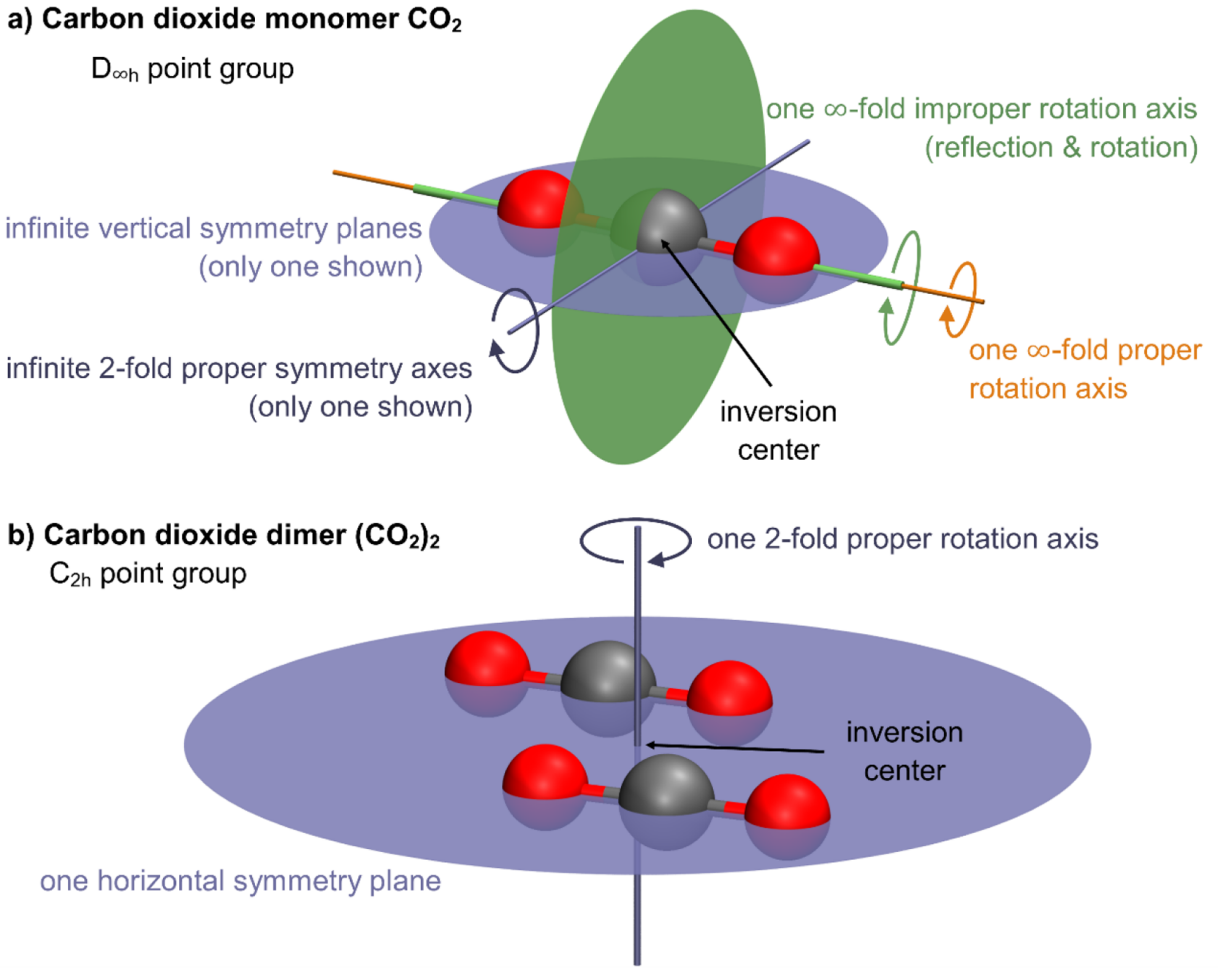
Symmetry elements of CO_2_ and (CO_2_)_2_. (a) CO_2_ in the *D*_∞*h*_ point group is highly symmetric. It contains an inversion center (black arrow), an infinite-fold improper rotation axis (green), and an infinite-fold proper rotation axis (yellow). There are infinite choices of degrees of rotation for the infinite-fold axes. Additionally, there is an infinite set of vertical symmetry planes and 2-fold proper rotation axes (blue). (b) In contrast, (CO_2_)_2_ in its equilibrium geometry, as a slipped-parallel structure of *C*_2*h*_ symmetry, contains only three symmetry elements: an inversion center (black arrow), a 2-fold rotation axis (blue), and a horizontal symmetry plane (blue). Due to its lower symmetry, it features more IR-active bands than the monomer.

The (CO_2_)_2_ dimer can be distinguished from the monomer by consideration of its structure and symmetry. The linear CO_2_ monomer (Figure 1a) comprises four normal modes (3*N* – 5 with *N* = 3) and is in the *D*_∞*h*_ point group. Only three modes are in the IR-active irreducible representation A_1u_ or E_1u_, namely, the antisymmetric stretch ν_3_(A_1u_) and the bending ν_2_(E_1u_). The bending occurs as two degenerate normal modes; hence, only two distinguishable IR-active vibrations are considered in total. Considering the structure of the (CO_2_)_2_ dimer, Kalugina et al.^22^ suggested the slipped-parallel 60–60–0 conformer as the global minimum. Other conformers are transition states (T-shaped and crossed dimer) or higher in energy (linear dimer). For the assignment of our MI-IR spectra, the consideration of the mentioned slipped-parallel dimer is sufficient; hence, we omit any anharmonic calculations of the other conformers.

The slipped-parallel (CO_2_)_2_ dimer (Figure 1b) is in the *C*_2*h*_ point group and has 12 vibrational modes (3*N* – 6 with *N* = 6). Six out of 12 modes are in the IR-active irreducible representation A_u_ or B_u_, namely, the in-phase antisymmetric stretch ν_9_(B_u_), the in-phase symmetric stretch ν_10_(B_u_), the in-phase out-of-plane bending ν_7_(A_u_), the in-phase in-plane bending ν_11_(B_u_), and two intermolecular modes ν_8_(A_u_) and ν_12_(B_u_). While the intermolecular modes are outside the atmospheric IR window in the far-infrared region,^28^ the remaining four are inside the atmospheric window. The ν_10_(B_u_) vibration in the atmospheric window is too weak to be detected in the present work. That said, an IR spectrum containing (CO_2_)_2_ dimers comprises three additional mid-IR bands compared to that of a pure monomer spectrum. In the following experimental spectra, we make use of these differences to elaborate on the impact of the (CO_2_)_2_ dimer spectral features relative to the CO_2_ monomer features.

## Results and Discussion

**Figure 2:**
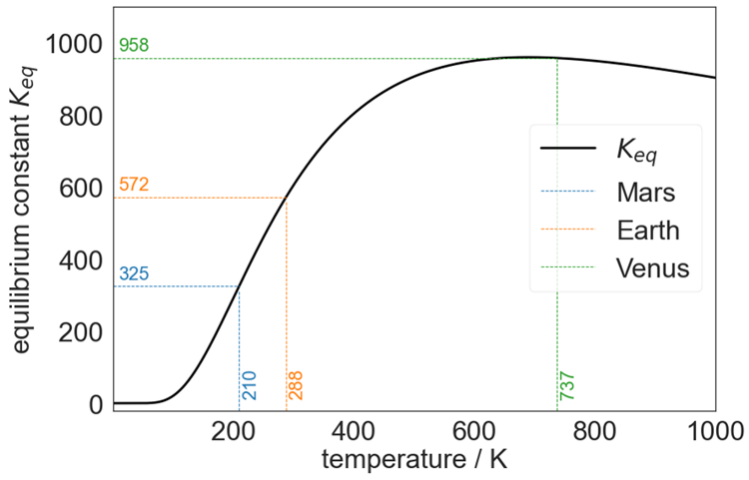
Temperature-dependent equilibrium constant *K*_eq_ for (CO_2_)_2_ → 2CO_2_ between 30 and 1000 K calculated within the rigid rotor harmonic oscillator (RRHO) approximation in the KiSTheIP program by Canneaux et al. (ref (21)) and relying on the electronic energy and the harmonic frequencies at the CCSD(T)-F12/VTZ-F12 level of theory.

Figure 2 depicts the calculated temperature dependence of the equilibrium constant *K*_eq_ for the (CO_2_)_2_ dimer dissociation reaction. For temperatures above 50 K, the monomer is thermodynamically favored (cf. thermochemistry data in Figure S1), and the equilibrium constant *K*_eq_ is on the CO_2_ monomer side. This is the case for various planetary atmospheres. While the equilibrium constant *K*_eq_ increases steeply for temperatures above 300 K (like Earth’s atmosphere), it reaches a maximum at around 700 K (like Venus’s atmosphere). The dimer dissociation, however, depends also on the partial pressure of carbon dioxide *p*(CO_2_) in the respective environment (cf. the pressure dependence of the degree of dissociation in Figure S2). On Earth and Mars, the partial pressure of carbon dioxide is very low (*p*(CO_2_) ≪ 1 bar), leading to high degrees of dissociation. On Venus, however, the high partial pressure (*p*(CO_2_) ∼ 90 bar) leads to a lower degree of dissociation α, although the high temperature favors dissociation.

For Venus, the very dense atmosphere composed of 96.5% carbon dioxide results in a significant fraction of dimers. According to our thermodynamic estimations, the ratio of monomers to dimers is approximately 12:1, resulting in a partial pressure of (CO_2_)_2_ dimers of 6.98 bar on Venus (7.6 vol %). Mars has an atmosphere that is composed of 95.1% carbon dioxide but is about 15 000 times thinner than that of Venus (6.36 mbar vs 92 bar). Consequently, the dimer is only a trace species on Mars, despite the much colder climate on Mars (210 vs 737 K). Hence, the monomer-to-dimer ratio is about 5.4 × 10^5^:1, and the (CO_2_)_2_ partial pressure is only 0.111 μbar (17 ppmv). By contrast to Mars and Venus, CO_2_ is only a trace species in our atmosphere (410 ppmv in a 1 bar atmosphere). This leads to a very high monomer-to-dimer ratio of 1.3 × 10^6^:1 and a (CO_2_)_2_ partial pressure of only 0.317 nbar (312 pptv) in the boundary layer above Earth’s surface at 288 K.

If (CO_2_)_2_ exists in the troposphere, then it is merely at a mixing ratio on the order of parts per trillion (ppt). At higher altitude, e.g., in the tropopause at ∼20 km or the mesopause at ∼80 km, temperatures are much lower, namely, about 200 and 150 K, respectively. Partial pressures are also lower, namely, by a factor of 10^2^ and 10^5^ at such altitudes. While the lower temperatures favor dimerization, the lower partial pressure disfavoring dimers is much stronger. This leads to much smaller dimer fractions in the higher layers of our atmosphere. Consequently, it is a huge challenge in terms of detection limit and sensitivity to spectroscopically observe (CO_2_)_2_ dimers on Mars and Earth. For comparison, our estimated mixing ratio of dimers (CO_2_)_2_ of 312 pptv on Earth is on the same scale as that of the OH radical^2^ or prominent ozone-depleting halocarbons, currently in between ∼80 pptv (CCl_4_) and ∼490 pptv (CFC-12) according to measurements at Manua Loa, Hawaii within the NOAA/GML *in situ* halocarbons program.

In both our neon matrix and the air-freezing experiments detailed below, we in fact do observe a considerable amount of (CO_2_)_2_ dimers. However, as we will also show below, the monomer/dimer ratio in our matrix experiments does not represent the ratio at 25 °C (or 65 °C). Rather than that, the gas precools to 40 K prior to being trapped in the matrix. Cooling of the gas from room temperature (or above) to 40 K shifts the equilibrium massively to the side of the dimers. That is, in our matrix experiment we trap an atmosphere enriched in dimers, where the monomer/dimer ratio is about 8:1 (see Table 1).

**Table 1:** Estimation of the (CO_2_)_2_ Partial Pressure and Monomer/Dimer Ratio^a^

	temp (K)	*K*_eq_	*p*CO_2_	*p*(CO_2_)_2_	ratio (monomer/dimer)
Mars	210	325	6.00 × 10^–3^ bar (∼95.1 vol %)	0.111 × 10^–6^ bar (∼17 ppmv)	54 157:1
Earth	288	572	0.426 × 10^–3^ bar (∼426 ppmv)	0.317 × 10^–9^ bar (∼312 pptv)	1 344 231:1
Venus	737	958	88.8 bar (∼96.5 vol %)	6.98 bar (∼7.6 vol %)	12:1
“matrix”	40	0.00273	0.400 × 10^–3^ bar	0.460 × 10^–6^ bar (∼46 ppmv)	8:1

a Equilibrium constants *K*_eq_ for a given temperature are taken from the calculation shown in Figure 2. Calculation of the dimer fraction *p*(CO_2_)_2_ and monomer/dimer ratio is detailed in the Supporting Information. The characteristic temperatures and carbon dioxide partial pressures *p*CO_2_ of Mars, Earth, and Venus are taken from ref (29).

**Figure 3:**
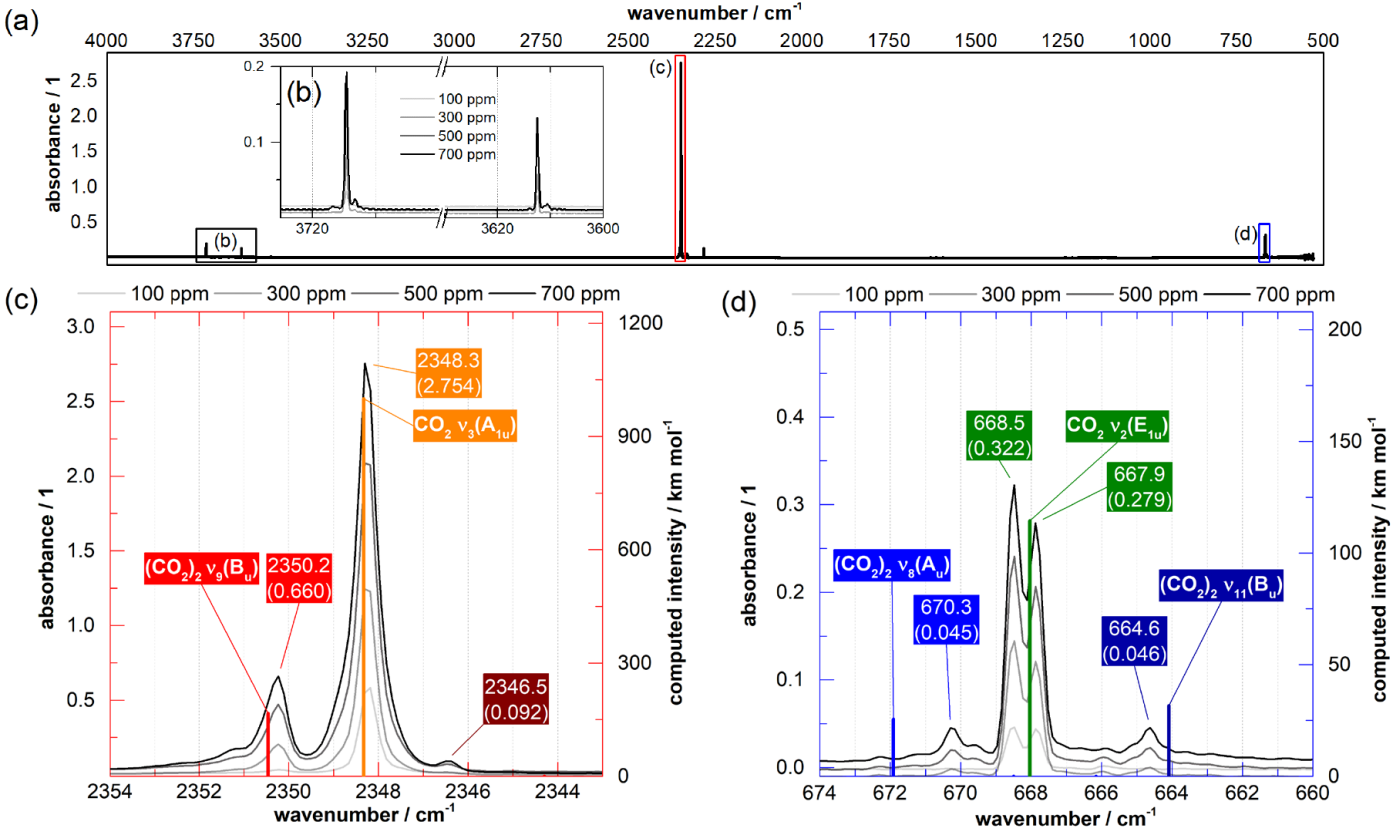
Matrix isolation of (CO_2_)_2_ dimers from gas-phase CO_2_/Ne mixtures at 6 K. (a) The mid-IR spectrum between 4000 cm^–1^ (2.5 μm) and 500 cm^–1^ (20 μm) exhibits three major absorption regions: (b) the Fermi resonance overtone region, which we do not consider in detail, (c) the antisymmetric stretch region, containing the (CO_2_)_2_ ν_9_(B_u_) transition, and (d) the bending region, containing the (CO_2_)_2_ ν_8_(A_u_) and ν_11_(B_u_) transitions. The assignment relies on frequencies calculated *in vacuo*, shown as colored lines. To better compare with the experiment, we scale all calculated frequencies by a factor of 0.9989 in panel c and 0.9924 in panel d and the calculated intensities by 0.2397.

Let us now turn to our experimental observations obtained for CO_2_/Ne mixtures after deposition as matrixes at 6 K. Figure 3a shows the IR spectra recorded in transmission–reflection for carbon dioxide mixing ratios ρ = 100–700 ppm. Other spectra for ρ = 700–4000 ppm are shown in Figures S4 and S5. At ρ = 100 ppm, the well-known CO_2_ monomer bands dominate the spectra. In the antisymmetric stretch region (Figure 3c), we observe the CO_2_ ν_3_(A_1u_) transition at 2348.3 cm^–1^. In the bending region (Figure 3d), we observe the CO_2_ ν_2_(E_1u_) transition as a doublet at 668.5 and 667.9 cm^–1^.^11^ Note that doublet splitting was observed in earlier studies for both the antisymmetric stretch and the bending region for carbon dioxide in argon and krypton matrixes but not in xenon, nitrogen, and deuterium matrixes.^30−36^ These doublets were identified as a matrix effect, reflecting that the monomer, of linear geometry, was in two different surroundings referred to as *matrix cages*. While we reproduced this splitting in argon matrixes, in neon matrixes we observe only a splitting for the CO_2_ ν_2_(E_1u_) transition.^11^ It has been shown that the matrix environment makes the two degenerate bending vibrations distinguishable, leading to the observed splitting of the band that corresponds to the bending vibration.^37−39^

For ρ ≥ 200 ppm, satellite bands appear, shifted from the assigned monomer bands by just a few wavenumbers. Specifically, two bands in the antisymmetric stretch region are shifted by +1.9 and −1.8 cm^–1^ compared to the CO_2_ ν_3_(A_1u_) transition (Figure 3c), and two bands in the bend region are shifted by +1.8/+2.4 and −3.3/–3.9 cm^–1^ compared to the CO_2_ ν_2_(E_1u_) transition (Figure 3d). As the shift between the satellite bands and the established monomer bands agrees with our VCI-calculated monomer–dimer shifts, we assign the satellite bands to the (CO_2_)_2_ dimer. Our VCI calculations of the slipped-parallel dimer in the *C*_2*h*_ point group reveal monomer–dimer shifts that are very similar to the experiment, namely, +2.2 cm^–1^ for the antisymmetric stretch and +3.9 and −3.9 cm^–1^ for the bend (cf. the line spectra in Figure 3, parts c and d). On the basis of these calculations, we assign the (CO_2_)_2_ bands as follows: the band at 2350.2 cm^–1^ to the (CO_2_)_2_ ν_9_(B_u_) transition and the bands at 670.3 and 664.6 cm^–1^ to the (CO_2_)_2_ ν_8_(A_u_) and ν_11_(B_u_) transitions, respectively.

The present VCI calculations systematically overestimate the experimental frequencies by approximately 5 cm^–1^. In parts c and d of Figure 3, we scale the VCI frequencies of the monomer in each the antisymmetric region by a factor of 0.9989 and the bending region by 0.9924 to match our neon MI-IR experiments. Consequently, we use the scaling factors derived from the monomer bands also for the dimer bands in the respective spectral regions. Table 2, Table S1, and Table S2 comprise the unscaled calculated frequencies, and we provide a variant of Figure 3 with unscaled calculated frequencies in Figure S3. The systematic overestimation of VCI frequencies compared to those of the MI-IR experiment is due to matrix effects^11^ and residual anharmonicity. Matrix effects could be computationally modeled by including the matrix atoms explicitly.^40^ In the present work, however, we rely on comparing shifts of bands rather than absolute wavenumbers. This strategy largely eliminates the impact of matrix effects on our comparison of experimental and computational IR spectra. As the matrix shift is very similar for dimers and monomers, it cancels out by inspecting differences in monomer and dimer band positions. For this reason, calculated and observed shifts between monomers and dimers (or similarly between conformers) are of crucial importance in the assignment. We have demonstrated this concept earlier for conformers of carbonic acid^17^ and monomers and dimers of carbonic acid hemiester^18,41^ trapped in noble gas matrixes.

**Table 2:** Monomer–Dimer Shift of the Stretching and Bending Regions^a^

	ref	(CO_2_)_2_ ν_9_(B_u_)	shift ←	CO_2_ ν_3_(A_1u_)	(CO_2_)_2_ ν_8_(A_u_)	shift ←	CO_2_ ν_2_(E_1u_)	shift →	(CO_2_)_2_ ν_11_(B_u_)
VCI	this study	2353.2	2.2	2351.0	677.0	3.9	673.1	–3.9	669.2
air (N_2_/Ar/O_2_)	this study	2350.9	2.2	2348.7	663.5	1.2	662.3		
neon	this study	2350.2	1.9	2348.3	670.3	1.8	668.5	–3.9	664.6
						2.4	667.9	–3.3	
nitrogen N_2_	(30)			2348.6	664.3	2.0	662.3	–1.7	660.6
argon	(30)	2345.8	1.3	2344.5	664.1	0.7	663.4	–3.5	659.9
						2.2	661.8	–2.0	
	(31)	2346.5	2.0	2344.5	664.2	0.8	663.4	–3.8	659.6
						2.4	661.8	–2.2	
	(36)	2346.7	2.7	2345.0	664.4	0.7	663.7	–3.8	659.9
						2.5	661.9	–2.0	
krypton	(31)	2342.5	2.0	2340.5	662.2	1.0	661.2	–2.6	658.6
						2.0	660.2	–1.6	
xenon	(31)	2336.0	1.5	2334.5	661.2	1.2	660.0	–1.5	658.5
deuterium D_2_	(33)			2344.0	666.3	1.2	665.1	–2.1	663.0

a Monomer–dimer shifts derived from absolute (unscaled) frequencies as assigned to the CO_2_ monomer and (CO_2_)_2_ dimer in various matrix-isolation experiments from literature and in the matrix-isolation experiments and VCI computations from this study. All values are in wavenumbers (cm^–1^).

Table 2 summarizes the monomer–dimer shifts, including our experiments and calculations as well as literature data. Our dimer assignment agrees with earlier observations of satellite bands in the IR spectroscopy of carbon dioxide in various matrixes.^30−36^ The frequency shifts due to dimerization are very systematic through all these experiments, which further confirms our assignment of the dimer bands. The upshifted band in the antisymmetric stretch region amounts to 1.3–2.7 cm^–1^ in argon, krypton, and xenon matrixes,^30−36^ 1.9 cm^–1^ in neon matrixes, and 2.2 cm^–1^ in the VCI calculation. The experimentally observed, downshifted band at 2346.5 cm^–1^ in the antisymmetric stretch region is not present in our calculations on CO_2_ and (CO_2_)_2_. We speculate that this band originates from larger (CO_2_)*_n_* clusters, where an assignment would require computations of the trimer (*n* = 3) or even higher oligomers (*n* ≥ 4). In previous MI-IR experiments, such downshifted bands have been assigned to higher oligomers of carbon dioxide.^30−36^ In the bending region, the upshifts are 0.7–2.5 cm^–1^ and the downshifts are −1.5 to −3.8 cm^–1^ in N_2_, Ar, Kr, Xe, and D_2_ matrixes,^30−36^ compared to +3.9 and −3.9 cm^–1^ in VCI calculations and +1.8/+2.4 and −3.3/–3.9 cm^–1^ in the neon matrix. In this context, we emphasize that wavenumbers in neon matrixes agree best with gas-phase spectra, i.e., the matrix effect in neon is the smallest one among all matrixes,^11^ which is why we decided to study the CO_2_ dimerization in neon matrixes.

The present VCI calculations allow for a correct assignment as they generally reproduce the positive monomer–dimer shift in the antisymmetric stretch region (Figure 3c) and one positive and one negative monomer–dimer shift in the bending region (Figure 3d). In the latter region, the calculated monomer–dimer shift from CO_2_ ν_2_(E_1u_) to (CO_2_)_2_ ν_11_(B_u_) agrees with the experiment, whereas the shift from CO_2_ ν_2_(E_1u_) to (CO_2_)_2_ ν_8_(A_u_) is slightly overestimated. Further improvement in our VCI calculations may be achieved by an extension of the multimode potential energy surface to couple more than three modes. However, we do not expect such calculations to improve the assignment; hence, we consider them as beyond the scope of the present work. Note that VSCF and VCI calculations including up to three mode couplings have been previously performed by Maystrovsky et al.^12^ Considering the mbCO2/grid/VCI calculations in ref (12), the monomer–dimer shift from CO_2_ ν_2_(E_1u_) to (CO_2_)_2_ ν_11_(B_u_) would be −4.5 cm^–1^, the shift from CO_2_ ν_2_(E_1u_) to (CO_2_)_2_ ν_8_(A_u_) would be +13.7 cm^–1^, and the shift from CO_2_ ν_2_(E_1u_) to (CO_2_)_2_ ν_11_(B_u_) would be +4.7 cm^–1^. These shifts do not agree with our or any previous MI-IR experiments,^30−36^ which is why we rely on our present VCI calculations for the final assignment. The discrepancy of the monomer–dimer shifts from ref (12) is most likely rooted in the VCI results of the dimer, where the authors correctly stress that the VCI eigenvectors are very sensitive to small changes in the computation^12^ and emphasize the value of additional investigations. The assigned monomer–dimer shifts observed from our and previous MI-IR experiments may be a reference for future VCI investigations.

In addition to the band positions, the band intensities are also of interest. In the region of 1400–1200 cm^–1^, we do not observe the (CO_2_)_2_ ν_10_(B_u_) transition. This agrees with the calculated IR intensities, where the intensity of ν_10_(B_u_) is 3–4 orders of magnitude weaker than the intensities of the other transitions, so we are not sensitive enough in our spectroscopy experiment to detect this very weak absorption. The observed intensities allow us to give a first estimate of the monomer/dimer ratio in our experiment. To match the monomer-to-dimer intensity ratio as observed for ρ = 700 ppm (black trace in Figure 3, parts c and d), the VCI-calculated intensities of the dimer bands in Figure 3, parts c and d, need to be scaled by 0.2397. In other words, for ρ = 700 ppm, the monomer is the dominant species with a ratio of 4:1 compared to the dimer. A better quantification of the monomer/dimer ratio from the experiment is possible by investigation of the band areas, as explained below. On the basis of our assignment, we proceed to the central task of the present study, namely, quantifying the additional IR absorption of the (CO_2_)_2_ dimers from the experimental spectrum itself.

On the basis of the assigned dimer and monomer bands, we retrieve the band areas by numeric integration, as described in the Supporting Information. Figure S4 details this numerical integration of the monomer and dimer bands. Figure S6 shows the absolute areas of monomer and dimer bands individually in Figure S6a, with relative areas given in Figure S6, parts b and c. This quantification enables us to (1) estimate the monomer/dimer ratio in the matrix experiment and (2) quantify the increase in IR absorption (and thus radiative forcing) by the occurrence of dimers. This estimate of the dimer abundance based on band areas is more rigorous and accurate than the rough estimate based on band intensities detailed above.

We may compare the monomer/dimer ratio from our experiment with the estimation we gave in Table 1 from our thermochemical calculation. For ρ = 400 ppm in our neon matrix experiment, we obtain a monomer-per-dimer ratio of 0.83/0.15 = 5.5 in the asymmetric stretch region or 0.87/0.13 = 6.7 in the bending region (cf. the relative dimer band areas in Figure S6b). For ρ = 400 ppm, our thermochemical calculation predicts a monomer/dimer ratio of 8 at a temperature of 40 K. This implies that the composition in the neon matrix does not correspond to the gas-phase composition at room temperature, but to the gas-phase composition at roughly 40 K. While ideally we would like to trap the ambient-temperature gas-phase composition “as is”, this is apparently not the case in the matrix-isolation studies. We explain this observation by gas-phase molecules bouncing back from the matrix instead of being trapped right away. The gas-phase molecules are precooled during this hitting of the matrix and bounce back before eventually being trapped, apparently to 40 K in our experimental setup.

**Figure 4:**
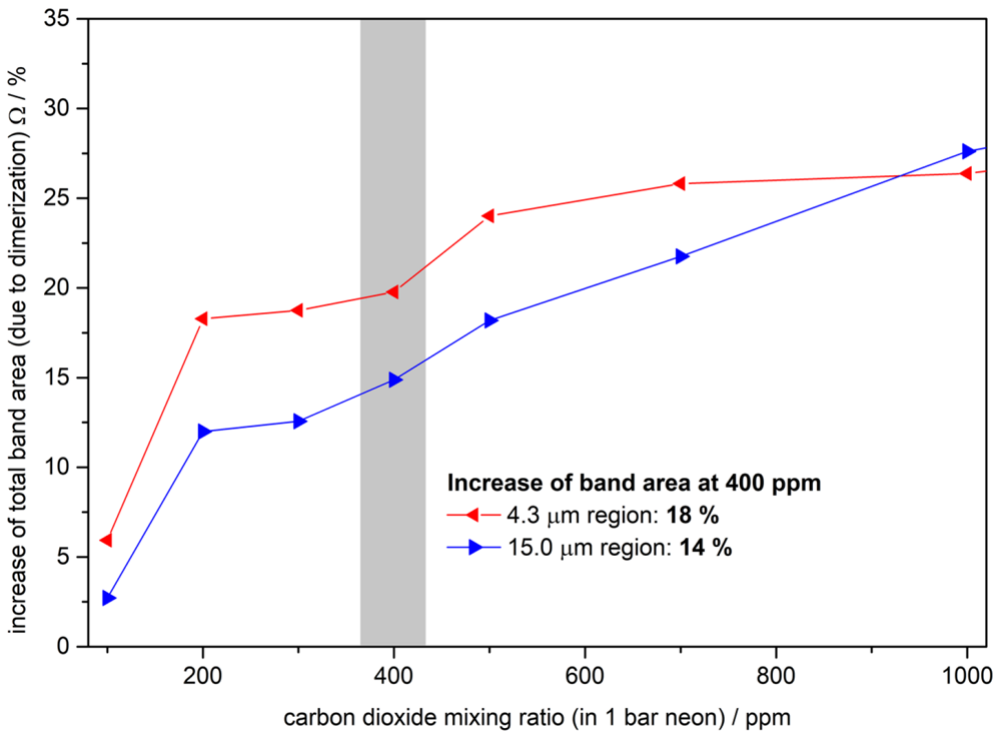
Amplification of the mid-IR absorption caused by (CO_2_)_2_ at different mixing ratios. The curve depicts how the overall mid-IR absorption is amplified due to additional dimer bands compared to that of the monomer alone. The red and blue curves are based on bands near 4.3 and 15.0 μm, respectively. Note that this graph applies to the precooled gas phase (presumably at 40 K) and is based on nonrotating molecules trapped in a neon matrix. The increase of the total band area due to dimerization is calculated by the relation Ω = .

The analysis of band areas also allows us to quantify the impact of dimers on radiative forcing and greenhouse warming. Assuming an equal absorption coefficient for the monomer and dimer, we can provide an estimate of how much the radiative forcing of carbon dioxide is increased by the occurrence of dimers. The (relative) dimer contribution to radiative forcing is then calculated from the increase of the total band area due to dimerization Ω = , where *a*(dim) is the area under the dimer bands and *a*(mono) is the area under the monomer band. Figure 4 shows Ω in red for the antisymmetric stretch and blue for the bending bands. For example, at ρ = 400 ppm Ω is 18% in the asymmetric stretch region. In general, both spectral regions provide two central observations: (1) Ω increases with increasing carbon dioxide mixing ratio ρ. (2) Ω increases steeply at lower ρ and less steeply at higher ρ (beyond 1000 ppm). In particular, Ω increases steeply from approximately 3–6% at ρ = 100 ppm to 15–20% at ρ = 400 ppm, and further to 22–26% at ρ = 700 ppm. Beyond ρ = 1000 ppm, Ω increases flatly from 25% to almost 50% at 4000 ppm.

**Figure 5:**
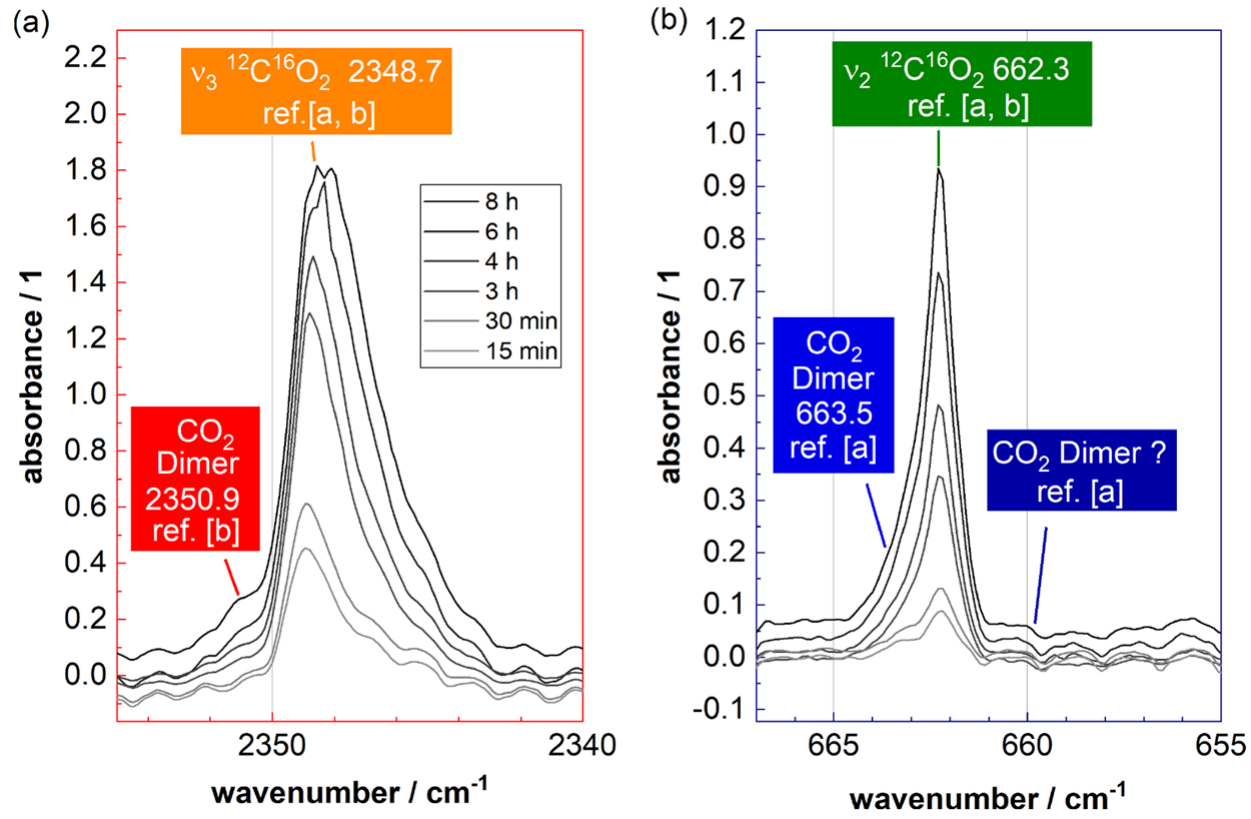
Matrix-isolation IR spectra of air (from Innsbruck on November 15, 2020; roughly 417 ppm of carbon dioxide) deposited at 15 K, where the main components of air act as the matrix material (N_2_/O_2_/Ar) and trace components are trapped as isolated molecules. The antisymmetric stretch region (a) and the bending region (b) of carbon dioxide are studied as a function of the deposition time (15 min, 30 min, 3, 4, 6, and 8 h). Assignment [a] refers to that of ref (30) and [b] to ref (36). Additional spectral regions are shown in the Supporting Information.

Our observation of significant absorption due to dimers in neon matrixes even at ρ = 400 ppm prompted us to study the MI-IR spectrum of ambient air. This was done by very slow deposition of air onto a cesium iodide window at 15 K, where the lab was well-vented with outdoor air right before the deposition. In this experiment the matrix is composed of air (N_2_/O_2_/Ar) itself, as opposed to neon in the mixing ratio series described above. Figure 5 depicts a deposition-time sequence of spectra in the antisymmetric stretch and bend regions. Figure S8 covers additional spectral regions, where we observe, besides CO_2_, bands arising from H_2_O, NO, and NO_2_ (most likely from traffic pollution) as well as clusters, e.g., (H_2_O)_2_ or CO_2_–N_2_, in the matrix. As shown in Table S3, the deviation of the band positions of these species from literature data measured in a N_2_ matrix is less than 1 cm^–1^.

Unlike the neon matrix spectra in Figure 3a–d, the mixing ratio of carbon dioxide in ambient air is constant (ρ = 417 ppm). Hence, we do not perform a mixing ratio series here, yet we increase the thickness of the air matrix with deposition time in Figure 5 to improve the signal-to-noise ratio. The interaction between CO_2_ and the matrix material *air* (N_2_/O_2_/Ar) is stronger than the interaction with the matrix material neon. This leads to a significant broadening of bands, a change of band intensities, and a more pronounced matrix shift. Despite the broadening, we see direct evidence for the presence of (CO_2_)_2_ in isolated air, namely, shoulders at 2350.9 and 663.5 cm^–1^. The shoulder assignment relies on the assignment of the monomer and dimer shown in Figure 3, parts c and d, as well as earlier literature on matrix isolation of carbon dioxide in nitrogen matrixes in ref (30) and in ref (36). Similar to the neon matrix spectra, the dimer bands are shifted by +2.2 and +1.2 cm^–1^ compared to the monomer bands. However, we do not observe the downshifted bending band as it is of very low intensity in N_2_ matrixes (and hence also in air matrixes).^30^ The downshifted band in the stretch region is missing, too. Thus, we assume that trimers and higher oligomers of CO_2_ are not present in the air matrix, as opposed to the neon matrix.

We have decomposed the bands shown in Figure 5 using three Gaussian functions to obtain more information on the monomer/dimer ratio in this matrix experiment. Figure S9 depicts this decomposition, where the black line represents the measured band, the red line the sum of three Gaussian functions, and the green lines the individual Gaussian peaks. To correctly reproduce the bending region, we used two Gaussian functions for the dimer and one for the monomer. For the stretch region, we need two Gaussian functions for the monomer, which is probably a consequence of the cage geometry: a cage splitting occurs for the monomer in the stretch region and a splitting for the dimer in the bending region. The splitting amounts to 1.3 and 0.4 cm^–1^, with very similar band areas and intensities for the Gaussian functions, indicating the cage splitting. In this analysis, each dimer band area is about 6–10 times smaller than the monomer band areas. We estimate that the IR absorption increases by about 13 ± 4% due to the dimers, which compares very well with the estimation from our neon experiments (see Figure 4). Also in this matrix experiment, the dimers are very likely present because the air is precooled before being trapped at the spectroscopy window. Upon precooling, a large fraction of monomers associate to produce dimers. The presence of such large fractions of carbon dioxide dimers in the air at ambient temperature is ruled out based on thermodynamics calculations.

## Conclusion

We here provide a systematic matrix-isolation study of carbon dioxide with increasing mixing ratios up to 4000 ppm. High-level VCI calculations that account for mode coupling and anharmonicity allow us to clearly identify monomer and dimer bands. As expected, while we barely observe any dimers at mixing ratios of 100 ppm, we observe a strong increase of the dimer fraction at higher mixing ratios. Similarly, we observe carbon dioxide dimers by trapping laboratory air (mainly N_2_/O_2_/Ar) containing approximately 417 ppm of CO_2_. On the basis of calculations of the equilibrium constants for the carbon dioxide monomer–dimer equilibrium, we conclude that the matrix composition represents the gas phase in thermodynamic equilibrium at 40 K. Accordingly, carbon dioxide is precooled before being trapped in the neon and air matrixes. Upon precooling, dimers and higher oligomers form. In the matrix experiment, the IR absorption due to carbon dioxide dimers is significant. The mid-IR absorption compared to that of monomers alone increases between 18% (at 400 ppm) and 35% (at 4000 ppm) due to dimerization. Although it is a rather weakly bound system, the (CO_2_)_2_ dimer can introduce additional mid-IR bands.

The fraction of carbon dioxide dimers in the current atmosphere of Earth (∼288 K) is in the parts-per-trillion range (see Table 1). In the coming 20 years, the carbon dioxide mixing ratio in the atmosphere will likely be doubled in comparison to preindustrial times and reach approximately 500 ppm. Although the increasing carbon dioxide mixing ratio will lead to a higher fraction of dimers, more sensitive methods will be necessary to identify dimers in Earth’s atmosphere spectroscopically. In contrast, we roughly estimate a 12:1 monomer/dimer ratio in the Venus atmosphere at 737 K and 88.8 bar of carbon dioxide. Consequently, climate models for Venus based on monomer bands alone would severely underestimate radiative forcing. Considering temperature and pressure broadening effects as the only mechanisms for IR band broadening^2^ would be insufficient. Hence, to properly account for band broadening, the occurrence of dimer satellite bands also ought to be considered. We tentatively propose that the (CO_2_)_2_ dimer is an underestimated driver for the Venus greenhouse effect that has previously not been recognized. On Earth and Mars, the (CO_2_)_2_ dimer fraction is too small to considerably increase IR absorption and act as a driver for the greenhouse effect of these planets.

## Supporting Information Available

Additional text explaining the results of the calculations and experiments in more detail, calculated frequencies of the monomer and dimer, experimentally observed bands in solid air, thermochemistry of the dimer dissociation, pressure dependence of the dissociation and dimer fraction, MI-IR spectra and calculated spectra without scaling, band integration of all MI-IR spectra of the dilution series in neon matrix (25 and 65 °C, gas phase), results of the band integration (absolute and relative area under the peak), heating experiment, complete MI-IR spectrum of isolated air, band integration of the MI-IR spectrum of isolated air, and additional references (PDF)

## References

[ref1] Anderson T. R.; Hawkins E.; Jones P. D. CO2, the Greenhouse Effect and Global Warming: From the Pioneering Work of Arrhenius and Callendar to Today’s Earth System Models. Endeavour 2016, 40 (3), 178–187. 10.1016/j.endeavour.2016.07.002. 27469427

[ref2] Wayne R. P. Chemistry of Atmospheres; Oxford University Press: New York, 2006.

[ref3] *Rise of carbon dioxide unabated*. NOAA Research News, June 4, 2020. https://research.noaa.gov/article/ArtMID/587/ArticleID/2636/Rise-of-carbon-dioxide-unabated (accessed 2020-12-01).

[ref4] *State of the Climate: Global Climate Report for Annual 2019*. NOAA National Centers for Environmental Information, January 2020. https://www.ncdc.noaa.gov/sotc/global/201913 (accessed 2020-12-01).

[ref5] O’Neill B. C.; Kriegler E.; Ebi K. L.; Kemp-Benedict E.; Riahi K.; Rothman D. S.; van Ruijven B. J.; van Vuuren D. P.; Birkmann J.; Kok K.; et al. The Roads Ahead: Narratives for Shared Socioeconomic Pathways Describing World Futures in the 21st Century. Glob. Environ. Chang. 2017, 42, 169–180. 10.1016/j.gloenvcha.2015.01.004.

[ref6] Huppmann D.; Rogelj J.; Kriegler E.; Krey V.; Riahi K. A New Scenario Resource for Integrated 1.5 °C Research. Nat. Clim. Chang. 2018, 8 (12), 1027–1030. 10.1038/s41558-018-0317-4.

[ref7] Tollefson J. How Hot Will Earth Get by 2100?. Nature 2020, 580 (7804), 443–445. 10.1038/d41586-020-01125-x. 32322083

[ref8] Wordsworth R.; Forget F.; Eymet V. Infrared Collision-Induced and Far-Line Absorption in Dense CO2 atm. Icarus 2010, 210 (2), 992–997. 10.1016/j.icarus.2010.06.010.

[ref9] Karman T.; Gordon I. E.; van der Avoird A.; Baranov Y. I.; Boulet C.; Drouin B. J.; Groenenboom G. C.; Gustafsson M.; Hartmann J. M.; Kurucz R. L.; et al. Update of the HITRAN Collision-Induced Absorption Section. Icarus 2019, 328 (March), 160–175. 10.1016/j.icarus.2019.02.034.

[ref10] Odintsova T. A.; Serov E. A.; Balashov A. A.; Koshelev M. A.; Koroleva A. O.; Simonova A. A.; Tretyakov M. Y.; Filippov N. N.; Chistikov D. N.; Finenko A. A.; et al. CO2–CO2 and CO2–Ar Continua at Millimeter Wavelengths. J. Quant. Spectrosc. Radiat. Transfer 2021, 258, 107400 10.1016/j.jqsrt.2020.107400.

[ref11] Dinu D. F.; Podewitz M.; Grothe H.; Loerting T.; Liedl K. R. Decomposing Anharmonicity and Mode-Coupling from Matrix Effects in the IR Spectra of Matrix-Isolated Carbon Dioxide and Methane. Phys. Chem. Chem. Phys. 2020, 22 (32), 17932–17947. 10.1039/D0CP02121K. 32744540

[ref12] Maystrovsky S.; Keçeli M.; Sode O. Understanding the Anharmonic Vibrational Structure of the Carbon Dioxide Dimer. J. Chem. Phys. 2019, 150 (14), 144302 10.1063/1.5089460. 30981225

[ref13] Bowman J. M. Beyond Platonic Molecules. Science (80-.) 2000, 290, 724 10.1126/science.290.5492.724. 11184203

[ref14] Oschetzki D.; Neff M.; Meier P.; Pfeiffer F.; Rauhut G. Selected Aspects Concerning the Efficient Calculation of Vibrational Spectra beyond the Harmonic Approximation. Croat. Chem. Acta 2012, 85 (4), 379–390. 10.5562/cca2149.

[ref15] Christiansen O. Vibrational Structure Theory: New Vibrational Wave Function Methods for Calculation of Anharmonic Vibrational Energies and Vibrational Contributions to Molecular Properties. Phys. Chem. Chem. Phys. 2007, 9 (23), 2942 10.1039/b618764a. 17551617

[ref16] Dinu D. F.; Podewitz M.; Grothe H.; Liedl K. R.; Loerting T. Toward Elimination of Discrepancies between Theory and Experiment: Anharmonic Rotational–Vibrational Spectrum of Water in Solid Noble Gas Matrices. J. Phys. Chem. A 2019, 123 (38), 8234–8242. 10.1021/acs.jpca.9b07221. 31433184 PMC6767348

[ref17] Bernard J.; Huber R. G.; Liedl K. R.; Grothe H.; Loerting T. Matrix Isolation Studies of Carbonic Acid - The Vapor Phase above the β-Polymorph. J. Am. Chem. Soc. 2013, 135 (20), 7732–7737. 10.1021/ja4020925. 23631554 PMC3663070

[ref18] Köck E.-M.; Bernard J.; Podewitz M.; Dinu D. F.; Huber R. G.; Liedl K. R.; Grothe H.; Bertel E.; Schlögl R.; Loerting T. Alpha-Carbonic Acid Revisited: Carbonic Acid Monomethyl Ester as a Solid and Its Conformational Isomerism in the Gas Phase. Chem. Eur. J. 2020, 26 (1), 285–305. 10.1002/chem.201904142. 31593601 PMC6972543

[ref19] Dinu D. F.; Podewitz M.; Grothe H.; Loerting T.; Liedl K. R. On the Synergy of Matrix-Isolation Infrared Spectroscopy and Vibrational Configuration Interaction Computations. Theor. Chem. Acc. 2020, 139 (12), 174 10.1007/s00214-020-02682-0. 33192169 PMC7652801

[ref20] Werner H. J.; Knowles P. J.; Manby F. R.; Black J. A.; Doll K.; Heßelmann A.; Kats D.; Köhn A.; Korona T.; Kreplin D. A.; et al. The Molpro Quantum Chemistry Package. J. Chem. Phys. 2020, 152 (14), 144107 10.1063/5.0005081. 32295355

[ref21] Canneaux S.; Bohr F.; Henon E. KiSThelP: A Program to Predict Thermodynamic Properties and Rate Constants from Quantum Chemistry Results. J. Comput. Chem. 2014, 35 (1), 82–93. 10.1002/jcc.23470. 24190715

[ref22] Kalugina Y. N.; Buryak I. A.; Ajili Y.; Vigasin A. A.; Jaidane N. E.; Hochlaf M. Explicit Correlation Treatment of the Potential Energy Surface of CO 2 Dimer. J. Chem. Phys. 2014, 140 (23), 234310 10.1063/1.4882900. 24952544

[ref23] Kats D.; Manby F. R. Communication: The Distinguishable Cluster Approximation. J. Chem. Phys. 2013, 139 (2), 021102 10.1063/1.4813481. 23862916

[ref24] Kats D. Communication: The Distinguishable Cluster Approximation. II. the Role of Orbital Relaxation. J. Chem. Phys. 2014, 141 (6), 061101 10.1063/1.4892792. 25134543

[ref25] Ziegler B.; Rauhut G. Rigorous Use of Symmetry within the Construction of Multidimensional Potential Energy Surfaces. J. Chem. Phys. 2018, 149 (16), 164110 10.1063/1.5047912. 30384678

[ref26] Ziegler B.; Rauhut G. Efficient Generation of Sum-of-Products Representations of High-Dimensional Potential Energy Surfaces Based on Multimode Expansions. J. Chem. Phys. 2016, 144 (11), 114114 10.1063/1.4943985. 27004869

[ref27] Rauhut G. Configuration Selection as a Route towards Efficient Vibrational Configuration Interaction Calculations. J. Chem. Phys. 2007, 127 (18), 184109 10.1063/1.2790016. 18020632

[ref28] Norooz Oliaee J.; Dehghany M.; Rezaei M.; McKellar A. R. W.; Moazzen-Ahmadi N. Five Intermolecular Vibrations of the CO2 Dimer Observed via Infrared Combination Bands. J. Chem. Phys. 2016, 145 (17), 174302 10.1063/1.4966146. 27825225

[ref29] Williams D. R. *NASA Planetary Fact Sheets*. NASA, January 28, 2016. https://nssdc.gsfc.nasa.gov/planetary/planetfact.html (accessed January 15, 2022).

[ref30] Fredin L.; Nelander B.; Ribbegård G. On the Dimerization of Carbon Dioxide in Nitrogen and Argon Matrices. J. Mol. Spectrosc. 1974, 53 (3), 410–416. 10.1016/0022-2852(74)90077-0.

[ref31] Guasti R.; Schettino V.; Brigot N. The Structure of Carbon Dioxide Dimers Trapped in Solid Rare Gas Matrices. Chem. Phys. 1978, 34 (3), 391–398. 10.1016/0301-0104(78)85181-7.

[ref32] Irvine M.; Mathieson J.; Pullin A. The Infrared Matrix Isolation Spectra of Carbon Dioxide. II. Argon Matrices: The CO2Monomer Bands. Aust. J. Chem. 1982, 35 (10), 1971 10.1071/CH9821971.

[ref33] Irvine M.; Pullin A. The Infrared Matrix Isolation Spectra of Carbon Dioxide. I. Deuterium Matrices : Identification of Bands Due to Carbon Dioxide Dimers. Aust. J. Chem. 1982, 35 (10), 1961 10.1071/CH9821961.

[ref34] Knoezinger E.; Beichert P. Matrix Isolation Studies of CO2 Clusters Emerging from Adiabatic Expansion. J. Phys. Chem. 1995, 99 (14), 4906–4911. 10.1021/j100014a006.

[ref35] Gómez Castaño J. A.; Fantoni A.; Romano R. M. Matrix-Isolation FTIR Study of Carbon Dioxide: Reinvestigation of the CO2 Dimer and CO2···N2 Complex. J. Mol. Struct. 2008, 881 (1–3), 68–75. 10.1016/j.molstruc.2007.08.035.

[ref36] Schriver A.; Schriver-Mazzuoli L.; Vigasin A. A. Matrix Isolation Spectra of the Carbon Dioxide Monomer and Dimer Revisited. Vib. Spectrosc. 2000, 23 (1), 83–94. 10.1016/S0924-2031(99)00087-9.

[ref37] Gartner T. A.; Barclay A. J.; McKellar A. R. W.; Moazzen-Ahmadi N. Symmetry Breaking of the Bending Mode of CO2in the Presence of Ar. Phys. Chem. Chem. Phys. 2020, 22 (37), 21488–21493. 10.1039/D0CP02674C. 32954395

[ref38] Barclay A. J.; McKellar A. R. W.; Moazzen-Ahmadi N. New Infrared Spectra of CO2 – Ne: Fundamental for CO2 – 22Ne Isotopologue, Intermolecular Bend, and Symmetry Breaking of the Intramolecular CO2 Bend. Chem. Phys. Lett. 2021, 779 (July), 138874 10.1016/j.cplett.2021.138874.

[ref39] Sode O.; Ruiz J.; Peralta S. Theoretical Investigation of the Vibrational Structure of the Ar–CO2 Complex. J. Mol. Spectrosc. 2021, 380, 111512 10.1016/j.jms.2021.111512.

[ref40] Bader F.; Lindic T.; Paulus B. A Validation of Cluster Modeling in the Description of Matrix Isolation Spectroscopy. J. Comput. Chem. 2020, 41 (8), 751–758. 10.1002/jcc.26123. 31804712

[ref41] Bernard J.; Köck E.-M.; Huber R. G.; Liedl K. R.; Call L.; Schlögl R.; Grothe H.; Loerting T. Carbonic Acid Monoethyl Ester as a Pure Solid and Its Conformational Isomerism in the Gas-Phase. RSC Adv. 2017, 7 (36), 22222–22233. 10.1039/C7RA02792C. 28603608 PMC5450006

