# Supplementary material for: Increase of Radiative Forcing through Midinfrared Absorption by Stable CO2 Dimers?
Source: J Phys Chem A. 2022 May 9;126(19):2966–75. doi: 10.1021/acs.jpca.2c00857 (PMC9125687; doi:10.1021/acs.jpca.2c00857)
Supplement: Supplementary file 1 — jp2c00857_si_001.pdf [file jp2c00857_si_001.pdf]

Supplementary Information for Publication

Increase of Radiative Forcing through Mid-IR Absorption by Stable CO_2 Dimers?

*Dennis F. Dinu [a,b,c], Pit Bartl [b], Patrick K. Quoika [a], Maren Podewitz [a,c], Klaus R. Liedl [a], Hinrich Grothe [c,] *, Thomas Loerting [b,] **

a) Institute of General, Inorganic and Theoretical Chemistry, University of Innsbruck, A-6020 Innsbruck, Austria
b) Institute of Physical Chemistry, University of Innsbruck, A-6020 Innsbruck, Austria
c) Institute of Materials Chemistry, TU Wien, A-1060 Vienna, Austria

* corresponding authors: thomas.loerting@uibk.ac.at, hinrich.grothe@tuwien.ac.at

Supplementary text, figures, and tables

Thermodynamic stability of the $(CO_2)_2$ dimer

Figure S1 shows that the free enthalpy of dimerization is steeply decreasing with temperature. At about 50 K, the dimerization becomes exergonic, which means that the monomer is thermodynamically favored. Above 50 K the equilibrium constant $K = \exp\left({-\Delta G}/{RT} \right)$ is > 1 (c.f. Figure 1), meaning that the monomer fraction in the gas's total composition must be higher than the dimer fraction. At very low temperatures (< 50 K) entropy favors the dimer. Above 50K the monomer starts to be the dominant species, and the equilibrium constant increases steeply. That said, we may not expect any significant number of $(CO_2)_2$ dimers for conditions such as on Earth's surface (288K), on Mars (210K) or even on Venus (737K). However, it has been proven that dimers do exist on Venus [S1]. This is due to the pressure dependence of the monomer-dimer equilibrium.

The pressure dependent degree of dissociation for a given equilibrium constant $K_{eq}(T)$ is:

$$\alpha(T,p) = \sqrt{\frac{K_{eq}(T)}{K_{eq}(T)\ +\ 4\ *\ {p}/{p_0}}}$$

As one may see from Figure S2, the mixing ratio of carbon dioxide on Venus with 88.8 bars is high enough so that $\alpha \approx 0.85$. Hence, we can expect a significant amount of $(CO_2)_2$ dimers on Venus, while for the other above-mentioned conditions, the mixing ratio is so low that $\alpha \approx 1.0$, meaning complete dissociation. We now may compute the dimer fraction $x_a(T,p)$ or monomer fraction $x_b(T,p)$:

$$x_a(T,p) = \frac{1 - \alpha(T,p)}{1 + \alpha(T,p)},\ x_b = \frac{2 \cdot \alpha}{1 + \alpha}.$$

Figure S2 shows the pressure dependency of the dimer fraction $x_a(T,p)$ for the three equilibrium constants $K_{eq}(T)$ we computed for Earth, Mars, and Venus. This allows us to estimate the actual dimer fraction $x_a(T,p)$ for the specific carbon dioxide partial pressure on these planets. Finally, we can estimate the partial pressure of the dimer

$$p(dimer) = x_a(T,p) * p(CO_2)$$

and the dimer-monomer ration by

$$dimer - monomer\ ratio = \frac{x_a}{x_b}.$$

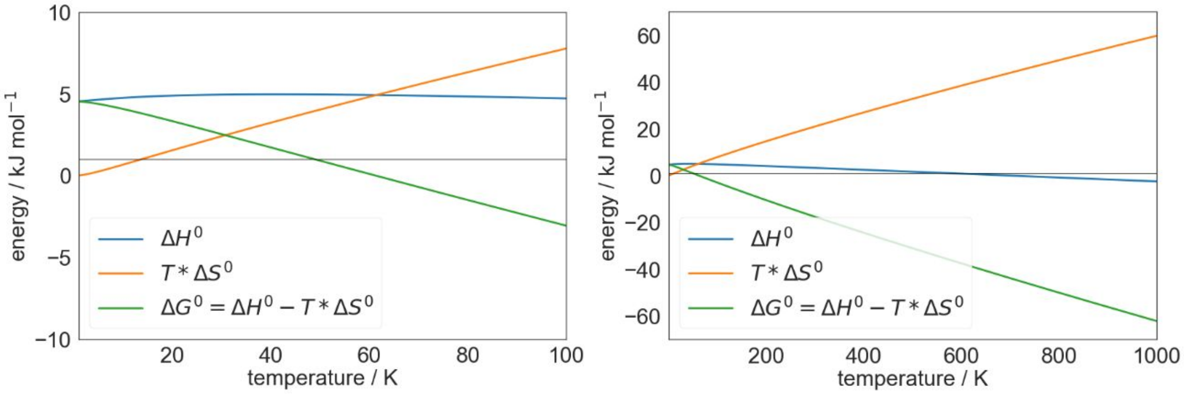

Figure S1. Thermochemistry of $(CO_2)_2 \leftrightarrow 2 * CO_2$ dissociation. Above temperatures of 50 K, the change of free enthalpy of dimerization ΔG^0 turns negative (left plot), mainly driven by the change of dimerization entropy $\mathrm{T} * \Delta S^0$, which becomes increasingly positive with increasing temperature. Consequently, the dissociation of the dimer is favored above 50 K. The temperature dependence of the free enthalpy of dimerization ΔG^0 is not perfectly linear when going to very high temperatures (right plot).

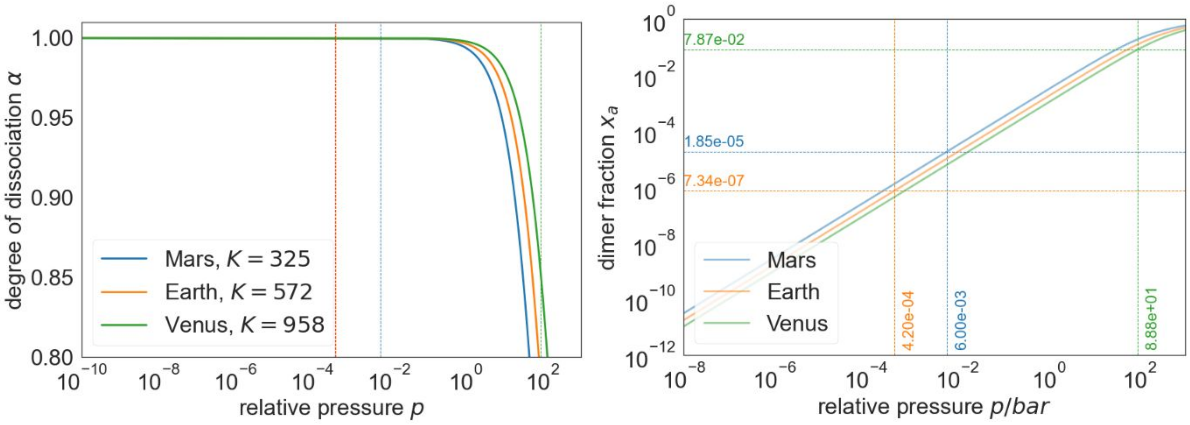

Figure S2. Pressure dependence of the degree of dissociation $\alpha(T,p)$ and dimer fraction $x_a(T,p)$. For partial pressures of carbon dioxide greater than 1 (= 10^0) bar, the degree of dissociation $\alpha(T,p)$ becomes significantly lower than 1, meaning that not all dimer is dissociating (left plot). Consequently, also the dimer fraction $x_a(T,p)$ depends on the partial pressure of carbon dioxide (right plot). This allows for an estimation of the dimer fraction. For example, on Earth (yellow) the partial pressure of carbon dioxide is 4.20*10^{-4} bar, leading to a dimer fraction of 7.34*10^{-7} and consequently to a partial pressure of the carbon dioxide dimer of 3.1*10^{-4} µbar mentioned in Table 1.

Anharmonic vibrational frequencies from the multi-mode PES based VSCF/VCI approach

In Figure 3, we employ scaling factors for both the frequencies and the intensities to better compare the computed data with the experimental data. On the one hand, these scaling factors are necessary to account for the frequency shifts introduced by the surrounding neon matrix, a purely physical phenomenon. On the other hand, these scaling factors also compensate for the small remaining anharmonicity deficit in our calculations, a purely numerical phenomenon. The latter is due to the multi-mode PES, which approximates the exact PES and allows for good results if converged. There are two major possibilities to converge the PES and improve the results: (1) Expand the multi-mode PES beyond 3-mode couplings. In theory, for $(CO_2)_2$ there could be up to 12-mode couplings involved in the PES. (2) Use a better method than DCSD-F12/VDZ-F12 for computing the electronic structure. Both improvements regarding the computational approach, however, are costly in terms of computational resources. Thus, we decided to accept slight discrepancies between the VSCF/VCI computed anharmonic frequencies and the "real" gas-phase frequencies.

Table S1. Calculated CO_2 monomer ($D_{\infty h}$) vibrational frequencies and IR intensities in cm^{-1}

		DCSD-F12/cc-pVDZ-F12 (used for assignment)				
**Mode**	**Label (Irrep)**	**Harmonic**	**VSCF**	**IR Intensity**	**VCI(SDTQ)**	**IR Intensity**
4	v_3 (A_{1u})	2399.04	2356.42	990.5	2351.03	1000.03
3	v_1 (A_{1g}), 2 v_2 (E_u) „fermi resonance"	1364.13	1352.3	0	1295.55 / 1399.55	0
2	v_2 (E_u)	677.09	674.15	57.73	673.13	57.76
1	v_2 (E_u)	677.08	674.15	57.73	673.13	57.76

Table S2. Calculated $(CO_2)_2$ dimer (C_{2h}) vibrational frequencies and IR intensities in cm^{-1}

		DCSD-F12/cc-pVDZ-F12 (used for assignment)				
**Mode**	**Label (Irrep)**	**Harmonic**	**VSCF**	**IR Intensity**	**VCI(SDTQ)**	**IR Intensity**
12	v_9 (B_u)	2400.64	2363.42	1913.88	2353.16	695.04
11	v_1 (A_g)	2395.68	2358.9	0	2346.99	0
10	v_2 (A_g)	1363.72	1350.06	0	1302.19	0.06
9	v_{10} (B_u)	1363.71	1350.89	0.25	1305.68	0.15
8	v_7 (A_u)	679.65	684.85	109.89	677.04	106.77
7	v_3 (A_g)	676.46	686.16	0	671.24	0
6	v_6 (B_g)	675.79	681.37	0	673.6	0
5	v_{11} (B_u)	674.14	683.91	138.59	669.16	131.62
4	v_4 (A_g)	104.02	115.03	0	98.93	0
3	v_5 (A_g)	45.32	50.84	0	42.9	0
2	v_8 (A_u)	27.58	67.42	0	45.46	0
1	v_{12} (B_u)	26.35	81.67	0.19	49.72	0.19
		**DCSD-F12/aug-cc-pVDZ**				
**Mode**	**Label (Irrep)**	**Harmonic**	**VSCF**	**IR Intensity**	**VCI(SDTQ)**	**IR Intensity**
12	v_9 (B_u)	2401.68	2363.97	1897.84	2354.01	765.63
11	v_1 (A_g)	2396.69	2362.35	0.00	2352.23	0.00
10	v_2 (A_g)	1364.05	1350.59	0.23	1307.85	0.13
9	v_{10} (B_u)	1363.99	1349.85	0.00	1303.90	0.00
8	v_7 (A_u)	682.79	687.20	109.54	679.39	107.02
7	v_3 (A_g)	679.99	688.45	0.00	673.63	0.00
6	v_6 (B_g)	679.24	683.67	0.00	675.86	0.00
5	v_{11} (B_u)	677.33	685.70	139.98	671.01	133.81
4	v_4 (A_g)	105.07	114.79	0.00	98.43	0.00
3	v_5 (A_g)	45.26	50.03	0.00	42.74	0.00
2	v_8 (A_u)	26.97	66.15	0.00	45.37	0.00
1	v_{12} (B_u)	21.28	79.94	0.20	48.27	0.21

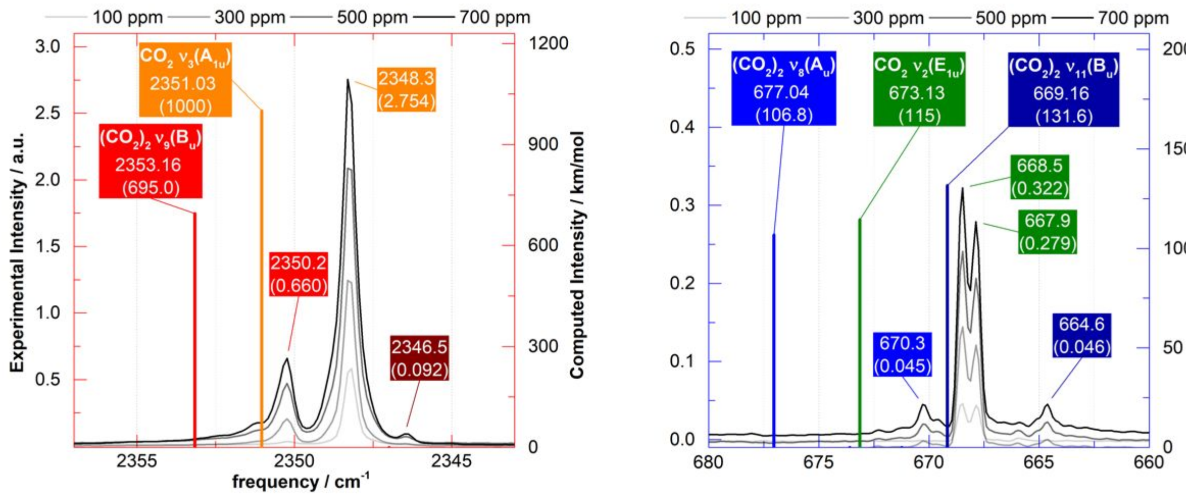

Figure S3. Matrix-Isolation of $(CO_2)_2$ dimers from gas-phase CO_2:Ne mixtures at 6 K.

(left) The antisymmetric stretch region (left), containing the $(CO_2)_2$ $\nu_9(B_u)$ transition and (right) the bending region, containing the $(CO_2)_2$ $\nu_8(A_u)$ and $\nu_{11}(B_u)$ transition. The calculated frequencies (colored lines) are not scaled, in contrast to Figure 3, which shows the same results including scaling factors.

Results from matrix isolation infrared (MI-IR) spectroscopy of carbon dioxide in neon

Numerical integration of the band areas in the Ne matrix spectra

In total, we evaluate 13 different mixing ratios of CO_2:Ne, being 1:10000 (100 ppm CO_2), 1:5000 (200 ppm CO_2), 1:3333 (300 ppm CO_2), 1:2500 (400 ppm CO_2), 1:2000 (500 ppm CO_2), 1:1429 (700 ppm CO_2), 1:1000 (1000 ppm CO_2), 1:500 (2000 ppm CO_2), 1:333 (3000 ppm CO_2), 1:250 (4000 ppm CO_2), 1:200 (5000 ppm CO_2), 1:133 (7500 ppm CO_2), 1:100 (10000 ppm CO_2). The marker bands were integrated using carefully chosen integration limits. The colors in all subsequent figures correspond to the color scheme used in Figure 3, Table 3, Table S1, Table S2 and Figure S3. Figure S4 and Figure S5 summarize the integration limits, which are maintained for all carbon dioxide mixing ratios. Above 4000 ppm, the intensity in the stretching region (2350 cm^{-1}) is too high for the detector, leading to unreasonable band-shapes. Thus, the stretching region was evaluated only up to 4000 ppm. We decided to include the evaluation at 3000 ppm and 4000 ppm, yet, this must be appreciated with care because also here we observe a band-splitting that may be due to the intensity overflow. Figure S4 depicts the integration for our main set of experiments, where the gas was mixed at 25°C, while Figure S5 comprises the second set of experiments, where the gas was mixed at 65°C.

(a)

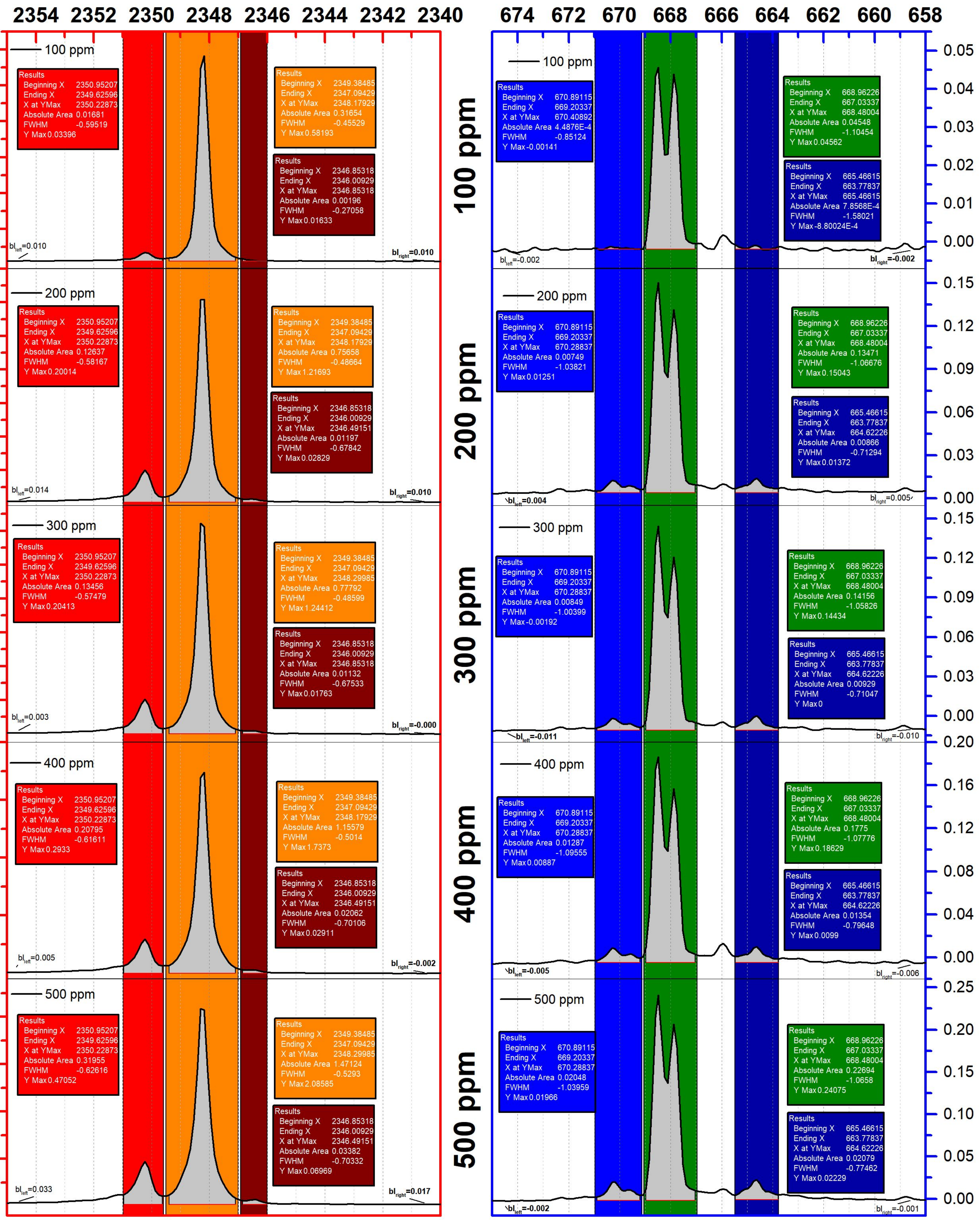
100 ppm
200 ppm
300 ppm
400 ppm
500 ppm
Results
Beginning X 2350.95207
Ending X 2349.62596
X at YMax 2350.22873
Absolute Area 0.01681
FWHM -0.59519
Y Max 0.03396
Beginning X 2349.38485
Ending X 2347.09429
X at YMax 2348.17929
Absolute Area 0.31654
FWHM -0.45529
Y Max 0.58193
Beginning X 2346.85318
Ending X 2346.00929
X at YMax 2346.85318
Absolute Area 0.00196
FWHM -0.27058
Y Max 0.01633
Beginning X 670.89115
Ending X 669.20337
X at YMax 670.40892
Absolute Area 4.4876E-4
FWHM -0.85124
Y Max -0.00141
Beginning X 668.96226
Ending X 667.03337
X at YMax 668.48004
Absolute Area 0.04548
FWHM -1.10454
Y Max 0.04562
Beginning X 665.46615
Ending X 663.77837
X at YMax 665.46615
Absolute Area 7.8568E-4
FWHM -1.58021
Y Max -8.80024E-4

S7

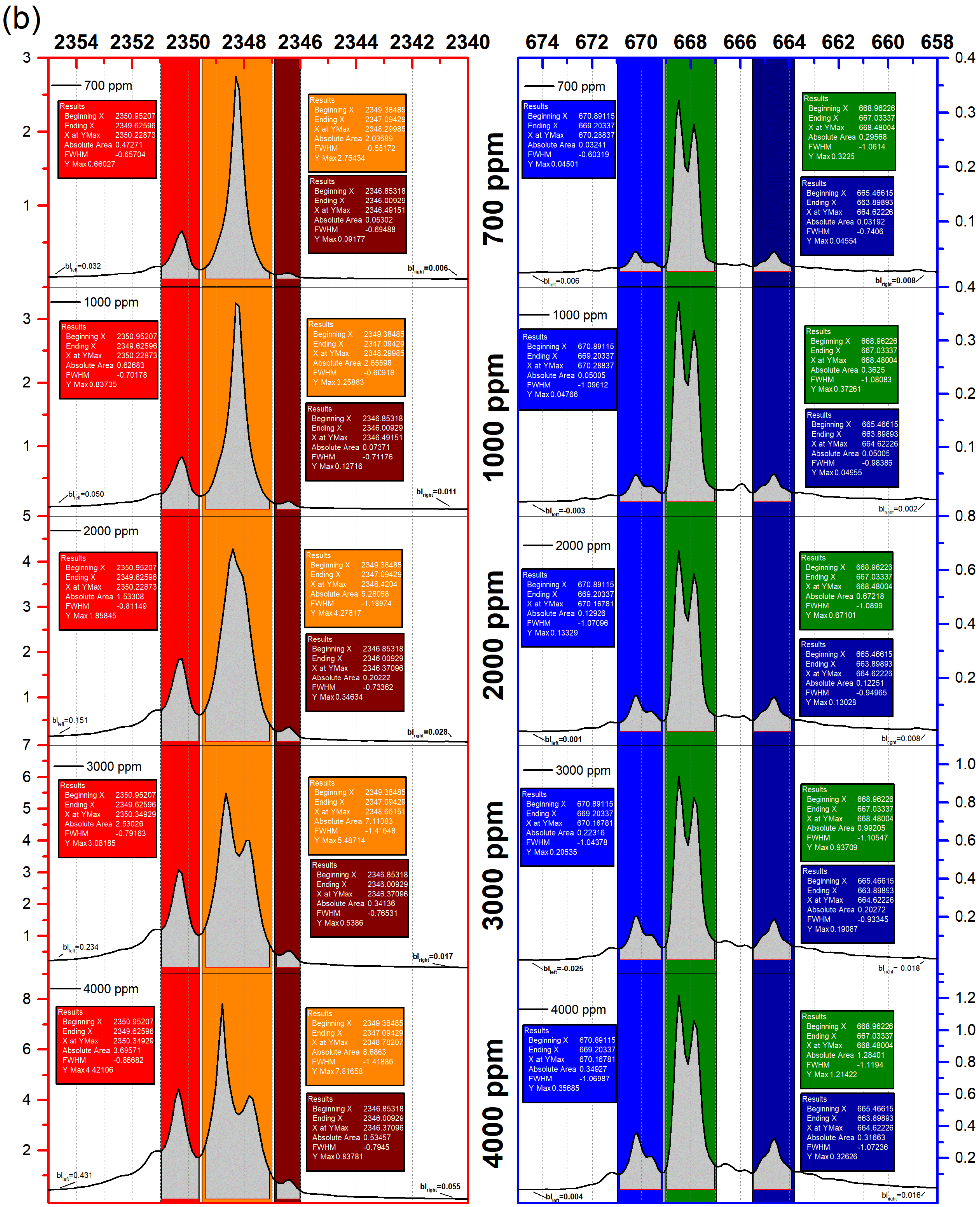

(b)
2354 2352 2350 2348 2346 2344 2342 2340
674 672 670 668 666 664 662 660 658
700 ppm
1000 ppm
2000 ppm
3000 ppm
4000 ppm
Results Beginning X 2350.95207 Ending X 2349.62596 X at YMax 2350.22873 Absolute Area 0.47271 FWHM -0.65704 Y Max 0.66027
Results Beginning X 2349.38485 Ending X 2347.09429 X at YMax 2348.29985 Absolute Area 2.03689 FWHM -0.55172 Y Max 2.75434
Results Beginning X 2346.85318 Ending X 2346.00929 X at YMax 2346.49151 Absolute Area 0.05302 FWHM -0.69488 Y Max 0.09177
bl left=0.032
bl right=0.006
Results Beginning X 670.89115 Ending X 669.20337 X at YMax 670.28837 Absolute Area 0.03241 FWHM -0.60319 Y Max 0.04501
Results Beginning X 668.96226 Ending X 667.03337 X at YMax 668.48004 Absolute Area 0.29568 FWHM -1.0614 Y Max 0.3225
Results Beginning X 665.46615 Ending X 663.89893 X at YMax 664.62226 Absolute Area 0.03192 FWHM -0.7406 Y Max 0.04554
bl left=0.006
bl right=0.008
Results Beginning X 2350.95207 Ending X 2349.62596 X at YMax 2350.22873 Absolute Area 0.62683 FWHM -0.70178 Y Max 0.83735
Results Beginning X 2349.38485 Ending X 2347.09429 X at YMax 2348.29985 Absolute Area 2.65598 FWHM -0.60918 Y Max 3.25863
Results Beginning X 2346.85318 Ending X 2346.00929 X at YMax 2346.49151 Absolute Area 0.07371 FWHM -0.71176 Y Max 0.12716
bl left=0.050
bl right=0.011
Results Beginning X 670.89115 Ending X 669.20337 X at YMax 670.28837 Absolute Area 0.05005 FWHM -1.09612 Y Max 0.04766
Results Beginning X 668.96226 Ending X 667.03337 X at YMax 668.48004 Absolute Area 0.3625 FWHM -1.08083 Y Max 0.37261
Results Beginning X 665.46615 Ending X 663.89893 X at YMax 664.62226 Absolute Area 0.05005 FWHM -0.98386 Y Max 0.04955
bl left=-0.003
bl right=0.002
Results Beginning X 2350.95207 Ending X 2349.62596 X at YMax 2350.22873 Absolute Area 1.53308 FWHM -0.81149 Y Max 1.85845
Results Beginning X 2349.38485 Ending X 2347.09429 X at YMax 2348.4204 Absolute Area 5.28058 FWHM -1.18974 Y Max 4.27817
Results Beginning X 2346.85318 Ending X 2346.00929 X at YMax 2346.37096 Absolute Area 0.20222 FWHM -0.73362 Y Max 0.34634
bl left=0.151
bl right=0.028
Results Beginning X 670.89115 Ending X 669.20337 X at YMax 670.16781 Absolute Area 0.12926 FWHM -1.07096 Y Max 0.13329
Results Beginning X 668.96226 Ending X 667.03337 X at YMax 668.48004 Absolute Area 0.67218 FWHM -1.0899 Y Max 0.67101
Results Beginning X 665.46615 Ending X 663.89893 X at YMax 664.62226 Absolute Area 0.12251 FWHM -0.94965 Y Max 0.13028
bl left=0.001
bl right=0.008
Results Beginning X 2350.95207 Ending X 2349.62596 X at YMax 2350.34929 Absolute Area 2.53026 FWHM -0.79163 Y Max 3.08185
Results Beginning X 2349.38485 Ending X 2347.09429 X at YMax 2348.66151 Absolute Area 7.11083 FWHM -1.41648 Y Max 5.48714
Results Beginning X 2346.85318 Ending X 2346.00929 X at YMax 2346.37096 Absolute Area 0.34136 FWHM -0.76531 Y Max 0.5386
bl left=0.234
bl right=0.017
Results Beginning X 670.89115 Ending X 669.20337 X at YMax 670.16781 Absolute Area 0.22316 FWHM -1.04378 Y Max 0.20535
Results Beginning X 668.96226 Ending X 667.03337 X at YMax 668.48004 Absolute Area 0.99205 FWHM -1.10547 Y Max 0.93709
Results Beginning X 665.46615 Ending X 663.89893 X at YMax 664.62226 Absolute Area 0.20272 FWHM -0.93345 Y Max 0.19087
bl left=-0.025
bl right=-0.018
Results Beginning X 2350.95207 Ending X 2349.62596 X at YMax 2350.34929 Absolute Area 3.69571 FWHM -0.86682 Y Max 4.42106
Results Beginning X 2349.38485 Ending X 2347.09429 X at YMax 2348.78207 Absolute Area 8.6863 FWHM -1.41886 Y Max 7.81658
Results Beginning X 2346.85318 Ending X 2346.00929 X at YMax 2346.37096 Absolute Area 0.53457 FWHM -0.7945 Y Max 0.83781
bl left=0.431
bl right=0.055
Results Beginning X 670.89115 Ending X 669.20337 X at YMax 670.16781 Absolute Area 0.34927 FWHM -1.06987 Y Max 0.35685
Results Beginning X 668.96226 Ending X 667.03337 X at YMax 668.48004 Absolute Area 1.28401 FWHM -1.1194 Y Max 1.21422
Results Beginning X 665.46615 Ending X 663.89893 X at YMax 664.62226 Absolute Area 0.31663 FWHM -1.07236 Y Max 0.32626
bl left=0.004
bl right=0.016

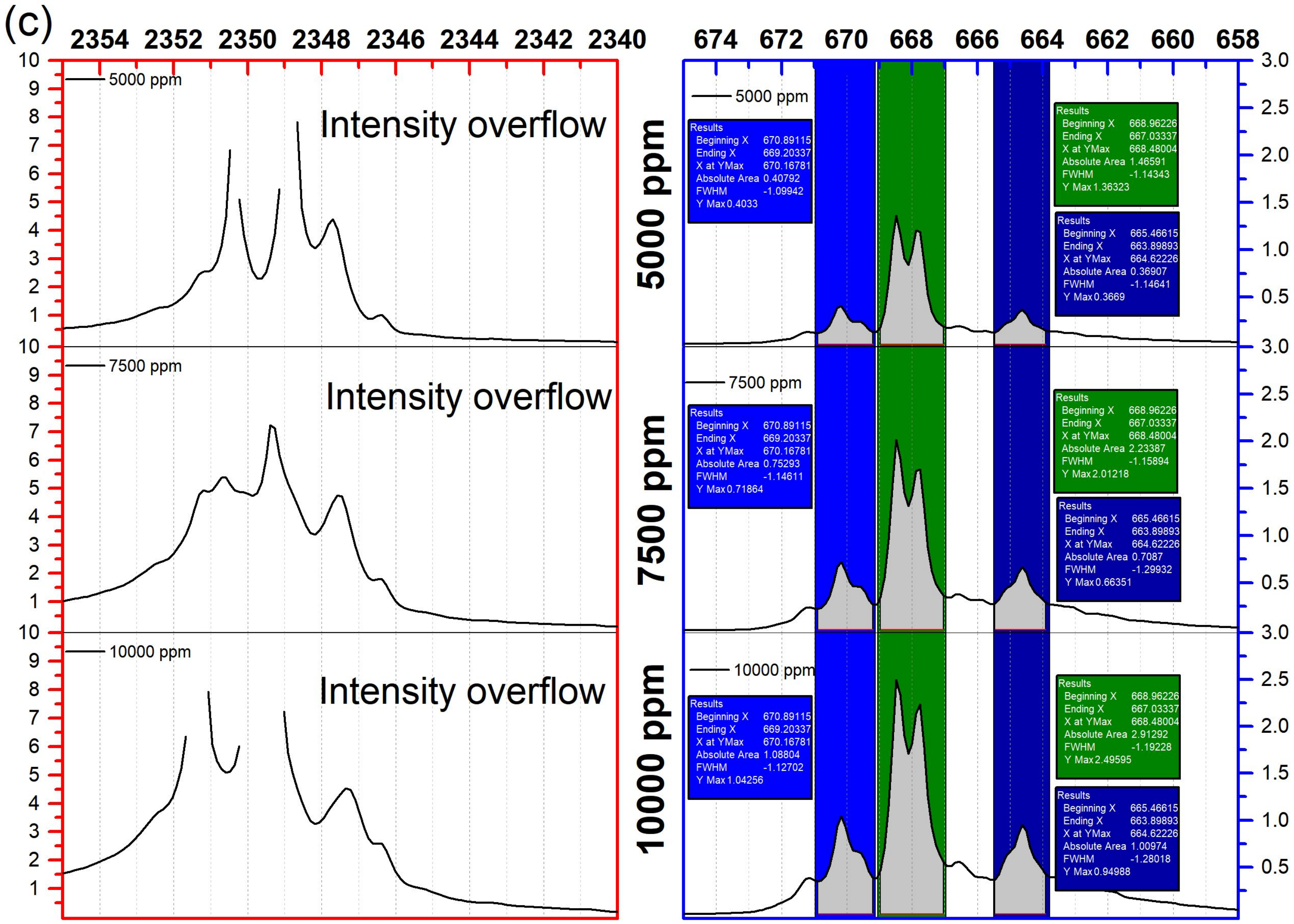

Figure S4. MI-IR spectra of CO_2:Ne mixtures containing (a) 100 ppm to 500 ppm, (b) 700 ppm to 4000 ppm, and (c) 5000 ppm to 10000 ppm carbon dioxide. The mixtures were equilibrated at 25°C and isolated at 6.0 K. The IR spectra were recorded at 6 K, with a resolution of 0.3 cm^{-1} using 512 scans.

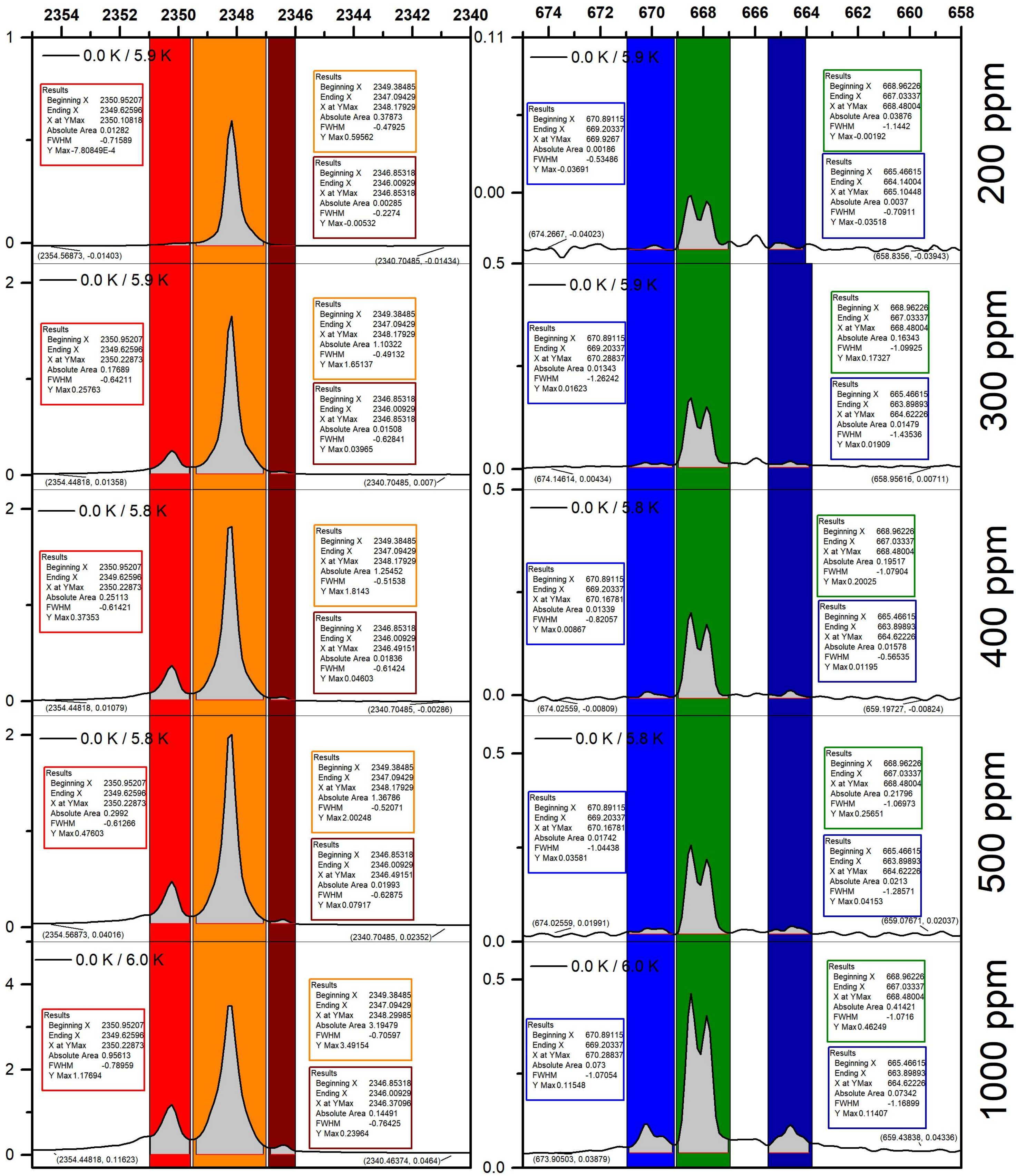

Figure S5. MI-IR spectra of CO_2:Ne mixtures containing 200 ppm to 1000 ppm carbon dioxide. The mixtures were equilibrated at 65°C and isolated at 6.0 K. The IR spectra were recorded at 6 K, with a resolution of 0.3 cm^{-1} using 32 scans.

Dimer contribution to the absorption cross-sections in the Ne matrix spectra

We here provide more details on the results of our numerical integration, specifically the evaluation of the dimer fraction $x(CO_2)_2$ and the relative increase of band area ΔA due to dimerization (c.f. Figure 4). We here elaborate on the absolute (Figure S6a) and relative (Figure S6b,c) areas of the individual marker bands by spectral regions. In the stretching region (Figure S6b, left) at 100 ppm carbon dioxide concentration, the CO_2 $v_3(A_{1u})$ transition at 2348.3 cm^{-1} amounts to 94.4% of the total band area, while the $(CO_2)_2$ $v_9(B_u)$ transition at 2350.2 cm^{-1} has 5.0%. In contrast, at 400 ppm, the monomer $v_3(A_{1u})$ transition at 2348.3 cm^{-1} has a contribution of 83.5% to the total area, while the dimer $v_9(B_u)$ transition at 2350.2 cm^{-1} has 15.0% (monomer-per-dimer ratio $\frac{0.83}{0.15} = 5.5$) . In other words, the contribution of the $(CO_2)_2$ bands is 3 times higher at 400 ppm than at 100 ppm carbon dioxide mixing ratio. At 1000 ppm the dimer band contribution increases merely to 18.7%. Hence, the dimer's contribution increases steeply at low mixing ratios and only marginally at high ratios. In the bending region (Figure S6b, right) at 100 ppm, the CO_2 monomer $v_2(E_{1u})$ transition at 668.5 cm^{-1} and 667.9 cm^{-1} has a contribution of 97.4%, while the $(CO_2)_2$ dimer $v_8(A_u)$ transition at 670.3 cm^{-1} has 1.0 % and the $(CO_2)_2$ dimer $v_{11}(B_u)$ transition at 664.6 cm^{-1} has 1.7 %. At 400 ppm, the monomer contributes 87.0%, while the two dimer bands contribute 12.9% in sum (monomer-per-dimer ratio $\frac{0.83}{0.15} = 6.7$). Consequently, the dimer bands' contribution is 4.5 times higher at 400 ppm than at 100 ppm. At 1000 ppm, the monomer and dimer bands amount to 78.4% and 21.6%, respectively. Consequently, the dimer bands' contribution is only 1.7 times higher at 1000 ppm than at 400 ppm carbon dioxide concentration. This observation confirms the picture seen in the stretching region. Figure S6b and S6c show the results for the gas mixing temperature of approximately 25°C and 65°C. The differences are most likely experimental discrepancies. Hence, we argue that within the chosen gas mixing temperature range of 25°C to 65°C there is no influence on the dimer fraction.

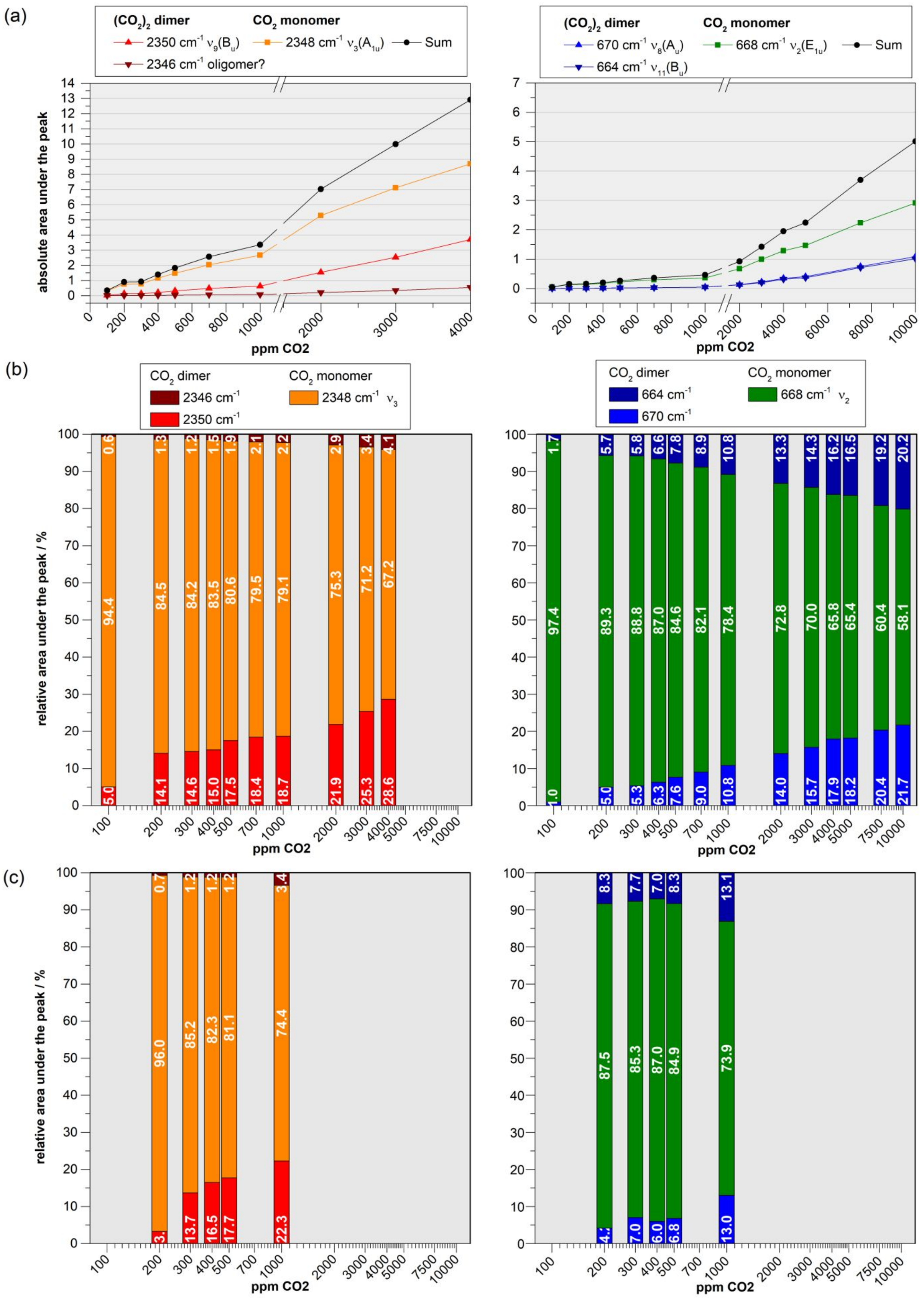

Figure S6. (a) Absolute and (b) relative area under the marker bands on a logarithmic ppm CO_2 scale, regarding CO_2:Ne gas mixtures at 25°C. (c) Relative area under the marker bands regarding CO_2:Ne gas mixtures at 65°C.

Heating of CO_2 trapped in Ne matrices

We mixed carbon dioxide (200 ppm, 300 ppm, 400 ppm, 500 ppm and 1000 ppm) with neon and equilibrated the mixtures at 65°C. For this purpose, the whole mixing apparatus was heated continuously. Each of the matrices was deposited at 5.8 K and then heated to 100 K. Figure S7 shows how the spectra change due to heating for 500 ppm CO_2. In the slow heating process from 5.8 K to 9.0 K the intensity ratio remains perfectly constant. This implies that the matrix is immobile, not allowing for cage-to-cage diffusion of CO_2 molecules. That is, CO_2 is indeed trapped – as desired for MI-IR experiments. At 9.5 K, the intensities start to decrease as a whole, implying sublimation of a significant fraction of neon, together with some CO_2. At 10 K, we observe a broad band, which signifies that practically all neon has sublimed, whereas some of the less volatile CO_2 remains on the sample holder. The broad band indicates an amorphous solid state, $(CO_2)_n$, where larger and larger clusters eventually develop to the amorphous solid. The amorphous CO_2 then remains upon continued heating to at least 30 K, as indicated by the broad and featureless band in Figure S7 between 10.0 and 30.2 K. At 40 K, features start to develop in this band, indicating the crystallization of the amorphous solid, where the features develop up to 80 K. At even higher temperatures the vapor pressure of crystalline CO_2 becomes too high so that the solid sublimes and is pumped off. This experiment demonstrates that the matrix is immobile at 6 – 9 K so that the composition of the gas-phase is retained in the neon matrix.

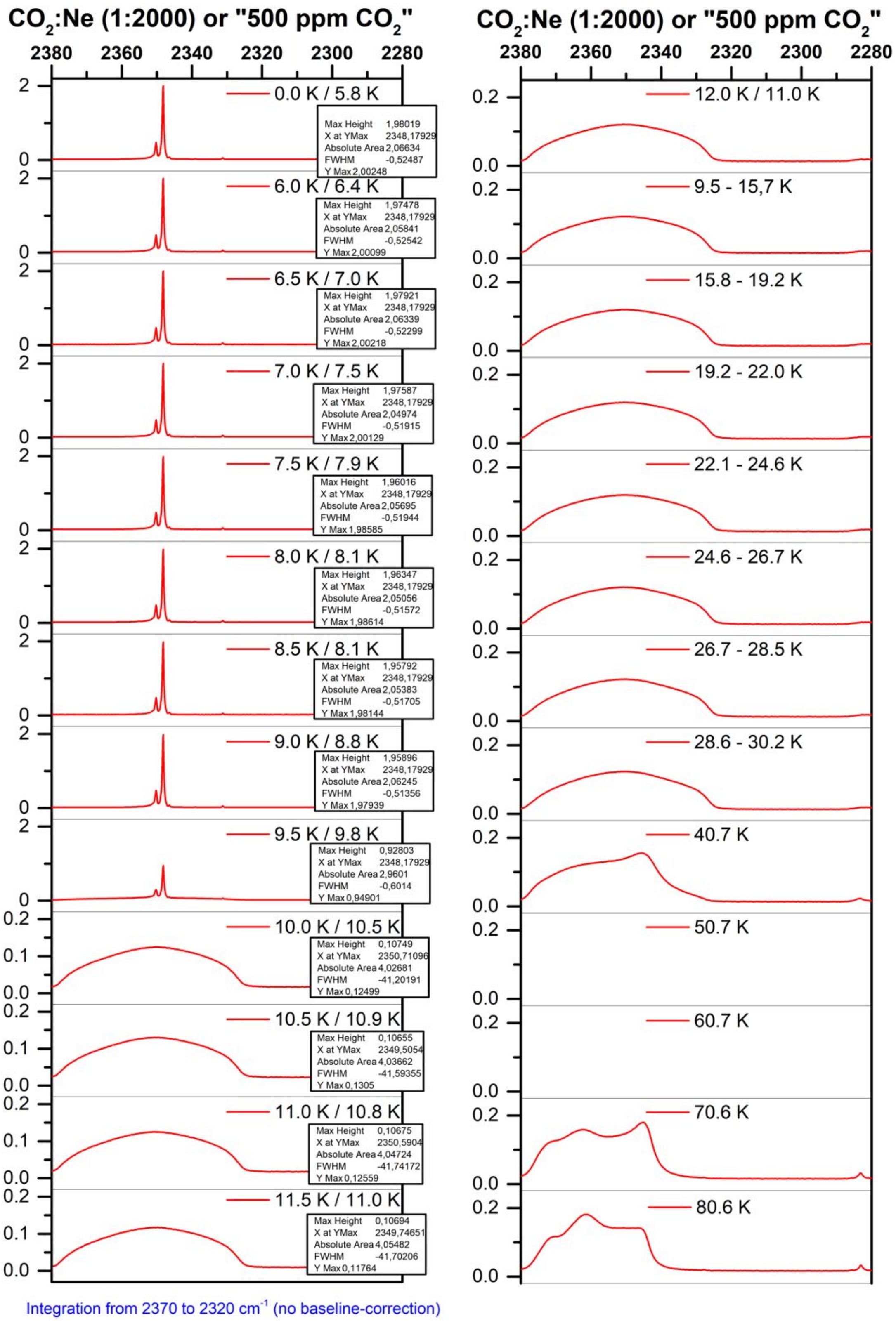

Figure S7. Heating experiment. 500 ppm CO_2 mixed with neon at 65°C and isolated at 6 K, then heated to the indicated temperatures.

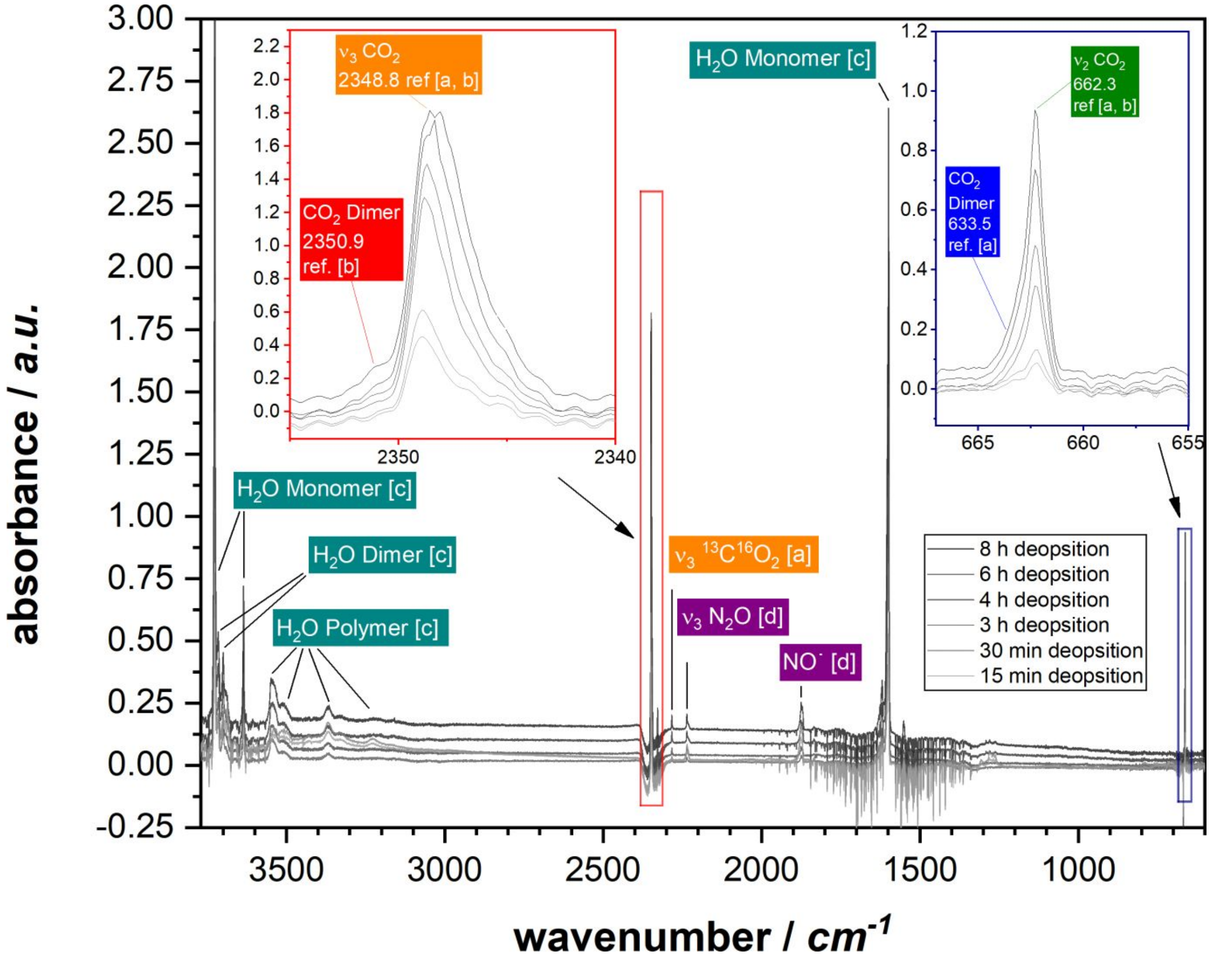

Figure S8. MI-IR spectra of air deposited at 15 K for deposition times of 15 min, 30 min, 3h, 4h, 6h and 8 h. Assignment of the peaks is presented in Table S3, where the indicated molecules are assigned based on [a] ref. [S2], [b] ref. [S3], [c] ref. [S4] and [d] ref. [S5].

Table S3: Mid-infrared bands of atmospheric trace gases trapped in solid air at 15 K (from the experiment shown in Figure S8). Reference values pertain to the nitrogen matrix.

Species	Wavenumber / cm^{-1}	Reference / cm^{-1}
H_2O Monomer	3726.5	3725.7 [S4]
H_2O Dimer	3715.5	3714.4 [S4]
H_2O Dimer	3699.5	3697.7 [S4]
H_2O Polymer	3688.9	3688 [S4]
H_2O Monomer	3633.9	3632.5 [S4]
H_2O Dimer	3546.6	3547.5 [S4]
H_2O Polymer	3511.2	3510 [S4]
ν_3 $(CO_2)_2$	2350.9	
ν_3 CO_2	2348.7	2348.6 [S2]
ν_3 $^{13}CO_2$	2283.2	2282.8 [S2]
ν_3 NO_2	2235.5	2235.55 [S5]
$NO^{\cdot}$	1874.5	1874.3 [S5]
H_2O Monomer	1598.5	1596.9 [S4]
ν_2 $(CO_2)_2$	663.7	
ν_2 CO_2	662.3	662.3 [S2]

Results of MI-IR spectroscopy of isolated air (carbon dioxide in nitrogen-oxygen-argon)

Assignment of all observed species in the spectra of isolated air

In the matrix isolation experiment of Innsbruck air at 15 K, we observed several bands (cf. Figure S8) that can be assigned easily based on literature matrix isolation experiments. Specifically, we identified multiple complexes of carbon dioxide, nitrogen, and water. Tursi et al. [S4] already assigned most of the water signals in a nitrogen matrix. In accordance with their work, we were able to assign the peaks that we measured to the respective water mono-, di- and polymers. Besides the bands for H_2O and the CO_2 bands described extensively in the manuscript, we also observed NO and NO_2 (cf. Table S3). The nitrogen oxides that we measured originated most likely from traffic pollution, which was always present due to the proximity of a much-frequented road (Innrain 52, Innsbruck, Austria on November 15, 2020). With the help of the work from Bahou et al. [S5], we are able to assign these species to the signals at 2235.5 (v_3 NO_2) cm^{-1} and 1874.5 cm^{-1} ($NO^{\cdot}$).

Dimer contribution to the absorption cross-sections in the air experiments

The bands in the isolated air spectrum are much broader than their counterparts in the Ne spectrum, but still are narrow enough to originate from single molecules. The broadness is owing to the more complex cages and the stronger interaction of the trapped species with N_2/O_2/Ar than with Ne. This made it more difficult to identify dimer and monomer bands. Yet, this was possible based on the calculations and the observations in Ne matrix. To disentangle monomer and dimer contributions to each band we used a Gaussian decomposition procedure. This procedure then also allowed us to retrieve band areas, just like in the Ne experiment. Again, by comparing the area of the decomposed peaks, we could estimate the fraction of $(CO_2)_2$ that is present in matrix-isolated Innsbruck air. The information is summarized in Figure S9). Similar to the conclusions from the Ne experiment we also found dimer band areas amounting to 15%.

This conclusion rests on a three-peak decomposition in the stretching region (see Figure S9), where the Gaussian centered at 2350.9 cm^{-1} pertains to the $(CO_2)_2$ dimer and the Gaussian at 2348.8 cm^{-1} and 2347.5 cm^{-1} to the v_3 mode of CO_2 monomers trapped in two different cage geometries. In the bending region, we were also able to identify three different Gaussians. Similar to the stretching region, it was not possible to decompose the peak into only two different peaks. The Gaussians at 663.5 cm^{-1} and 663.1 cm^{-1} originate from the $(CO_2)_2$ dimer and the one at 662.3 cm^{-1} from the v_2 CO_2 monomer signal. Those peaks have already been described by [S2] for pure nitrogen matrices, except for the one at 663.1 cm^{-1}. Noticing the proximity to the known dimer band and the same intensity of the Gaussians at 663.5 cm^{-1} and 663.1 cm^{-1} hints to an assignment as dimer band, where the small shift of 0.4 cm^{-1} is again most likely caused by differing cage geometries.

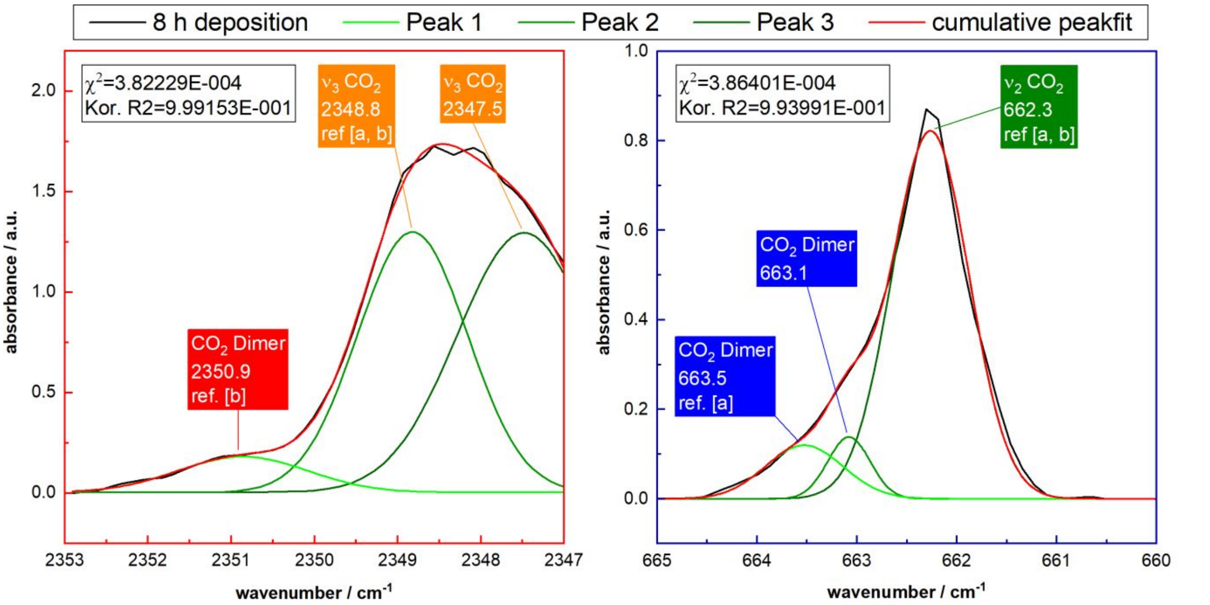

Peakindex	Peak type	FWHM / cm^{-1}	Max. Height	Center / cm^{-1}	Peak Area
1	Gaussian	1.84	0.17	2350.9	7.93
2	Gaussian	1.56	1.29	2348.8	48.9
3	Gaussian	1.9	1.29	2347.5	43.17

Peakindex	Peak type	FWHM / cm^{-1}	Max. height	Center / cm^{-1}	Peak area
1	Gaussian	0.92	0.12	663.5	11.78
2	Gaussian	0.5	0.14	663.1	7.46
3	Gaussian	0.91	0.82	662.3	80.76

Figure S9. Gaussian decomposition of the v_2 and v_3 peak of carbon dioxide in isolated air. The assignment corresponds to what is observed in pure nitrogen [a] in ref. [S2] and [b] in ref. [S3].

Supplementary References

(S1) Asfin, R. E.; Buldyreva, J. V.; Sinyakova, T. N.; Oparin, D. V.; Filippov, N. N. Communication: Evidence of Stable van Der Waals CO2 Clusters Relevant to Venus Atmosphere Conditions. *J. Chem. Phys.* **2015**, *142* (5).

(S2) Fredin, L.; Nelander, B.; Ribbegård, G. On the Dimerization of Carbon Dioxide in Nitrogen and Argon Matrices. *J. Mol. Spectrosc.* **1974**, *53* (3), 410–416.

(S3) Schriver, A.; Schriver-Mazzuoli, L.; Vigasin, A. A. Matrix Isolation Spectra of the Carbon Dioxide Monomer and Dimer Revisited. *Vib. Spectrosc.* **2000**, *23* (1), 83–94.

(S4) Tursi, A. J.; Nixon, E. R. Matrix-Isolation Study of the Water Dimer in Solid Nitrogen. *J. Chem. Phys.* **1970**, *52* (3), 1521–1528.

(S5) Bahou, M.; Schriver-Mazzuoli, L.; Camy-Peyret, C.; Schriver, A.; Chiavassa, T.; Aycard, J. P. Infrared Matrix Spectra of the N2O ··· O2 Complex in Solid Nitrogen. The N2O ··· O2 + O Thermal Diffusion Limited Reaction. *Chem. Phys. Lett.* **1997**, *265* (1–2), 145–153.
